# Chemically Induced Chromosomal Interaction (CICI) method to study chromosome dynamics and its biological roles

**DOI:** 10.1038/s41467-022-28416-3

**Published:** 2022-02-09

**Authors:** Manyu Du, Fan Zou, Yi Li, Yujie Yan, Lu Bai

**Affiliations:** 1 Department of Biochemistry and Molecular Biology, The Pennsylvania State University, University Park, PA 16802 USA; 2 Center for Eukaryotic Gene Regulation, The Pennsylvania State University, University Park, PA 16802 USA; 3 Department of Physics, The Pennsylvania State University, University Park, PA 16802 USA

**Keywords:** Biophysics, Cell biology, Genetics, Genetic engineering

## Abstract

Numerous intra- and inter-chromosomal contacts have been mapped in eukaryotic genomes, but it remains challenging to link these 3D structures to their regulatory functions. To establish the causal relationships between chromosome conformation and genome functions, we  develop a method, Chemically Induced Chromosomal Interaction (CICI), to selectively perturb the chromosome conformation at targeted loci. Using this method, long-distance chromosomal interactions can be induced dynamically between intra- or inter-chromosomal loci pairs, including the ones with very low Hi-C contact frequencies. Measurement of CICI formation time allows us to probe chromosome encounter dynamics between different loci pairs across the cell cycle. We also conduct two functional tests of CICI. We perturb the chromosome conformation near a DNA double-strand break and observe altered donor preference in homologous recombination; we force interactions between early and late-firing DNA replication origins and find no significant changes in replication timing. These results suggest that chromosome conformation plays a deterministic role in homology-directed DNA repair, but not in the establishment of replication timing. Overall, our study demonstrates that CICI is a powerful tool to study chromosome dynamics and 3D genome function.

## Abstract (web summary)

Methods to selectively manipulate specific long-distance chromosomal interactions are limited. Here the authors develop a method called Chemically Induced Chromosomal Interaction (CICI) to engineer interactions and demonstrate that 3D conformation plays a causal role in establishing donor DNA preference during DNA repair.

## Introduction

3D chromosome conformation is thought to play critical roles in various nuclear processes, including DNA replication^1,2^, repair^3–5^, and transcription regulation^6–10^. Recent applications of the Chromosome Conformation Capture techniques (3C, 4C, Hi-C, etc.) have discovered numerous intra- and inter-chromosomal interactions^11–14^. We currently have a limited understanding of the functions of these long-distance chromosomal interactions. Many studies have examined the correlations between chromosome organization and genomic processes, but it remains challenging to investigate their causal relationships.

Toward this goal, the traditional genetic approach is to identify a protein that establishes the chromosomal interaction, mutate the protein, and probe the biological consequences. Unfortunately, some of these proteins, like cohesin and CTCF, are essential for maintaining the overall 3D structure of the chromosomes, and mutations of these proteins will generate global chromosomal changes that lead to pleiotropic effects. In addition, proteins that mediate chromosome interactions may have diverse functions. Mutations of these proteins may result in consequences that are not related to the change in 3D genome.

To elucidate the function of 3D chromosome without the confounding factors above, we need a method that allows us to selectively perturb chromosomal interactions. A number of solutions have been presented in recent years, including artificial tethering of chromosomes through tetrameric LacI^15,16^, forced chromosome looping by tethering dimerization factors with zinc-finger proteins^17^ or dCas9^18–20^. However, the applications of these techniques so far have been largely limited to well-studied enhancer-promoter loci like the LCR-β-globin region, which has the tendency to form loops. Some of these methods rely on constitutive protein–protein interactions, and therefore the interactions they generate are not dynamically controlled. Here, we (1) develop a method that can rapidly induce chromosomal interactions between different pairs of targeted loci, including ones with very low contact frequency, (2) measure the rate and efficiency of Chemically Induced Chromosomal Interactions (CICI) formation, and investigate their relations to the Hi-C contact frequencies of the targeted loci pair, (3) use time-lapse assay to monitor CICI formation and disruption in single cells in real time, and evaluate the dynamics of these events at different cell cycle stages, (4) perturb the chromosome conformation near a DNA double-stranded break and probe its impact on DNA repair, and (5) force interactions between early and late-firing replication origins and measure the resulting DNA replication timing. These studies demonstrate that CICI is a powerful tool to study chromosome dynamics and 3D genome functions.

## Results

### Implementation of Chemically Induced Chromosomal Interactions (CICI)

**Fig. 1:**
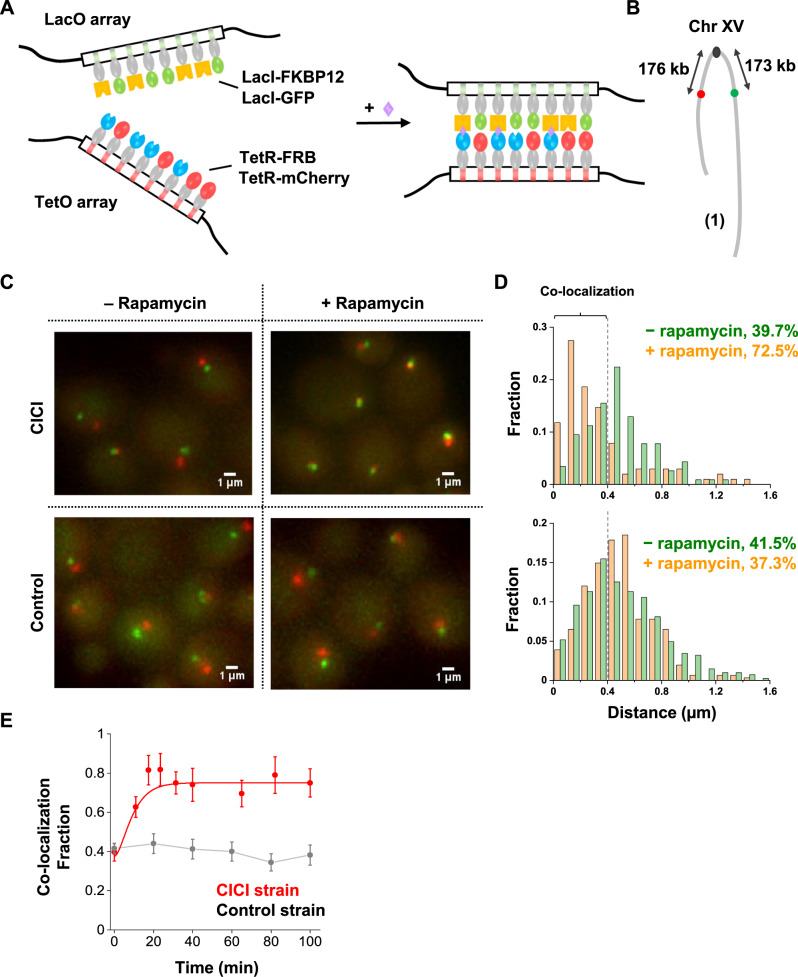
Implementation of CICI. **A** The scheme of CICI. LacO and TetO arrays were inserted into two genomic loci. Fusion proteins LacI-FKBP12 and TetR-FRB associated with these arrays dimerize in the presence of rapamycin. LacI-GFP and TetR-mCherry were also included for visualization. **B** LacO (green dot) and TetO (red dot) arrays are integrated 150 kb from the centromere on the left and right arms of Chr XV. **C** Visualization of the CICI strain (containing the complete CICI system in (**A**)) and the control strain (containing LacI and TetR instead of LacI-FKBP12 and TetR-FRB) ± rapamycin. **D** Histograms of distances between the two dots in the CICI strain (upper panel) and in the control strain (lower panel) ± rapamycin. The +rapamycin histogram includes measurements where the CICI or control strains were incubated with rapamycin for 1–2 h. In total, 116, 102, 407, and 308 cells were measured, respectively. The co-localization fraction in each condition is shown in the plots. **E** Fraction of co-localization as a function of rapamycin incubation time in the CICI (red) or the control strain (black). The total numbers of data points were 436 for CICI and 912 for control. Data are presented as mean values ± SEM. Source data for **D** and **E** are provided as a Source Data file.

The method we developed, CICI, involves two existing techniques, chemically induced dimerization (CID)^21,22^ and fluorescent repressor operator systems (FROS)^23,24^. In a commonly used CID system, FK506 binding protein (FKBP12) and the FKBP-rapamycin binding domain (FRB), two otherwise non-interacting factors, bind strongly in the presence of rapamycin^25^. We expressed LacI-FKBP12 and TetR-FRB fusion proteins in a rapamycin-resistant yeast strain containing LacO and TetO repeats at desired genomic locations (Fig. 1A). In addition to the FKBP12 and FRB fusions, we also introduced LacI-GFP and TetR-mCherry into the same strain for visualization. We expected the LacI-FKBP12 and TetR-FRB to bind tightly in the presence of rapamycin, resulting in the co-localization of the LacO and TetO “chromosome dots” (Fig. 1A). Note that if we use LacI-FKBP12-GFP and TetR-FRB-mCherry triple fusions, co-localized chromatin dots may represent two chromatin loci bound together as intended by CICI, or chromatin-bound FKBP12/FRB associated with free FRB/FKBP12. In order to have an unambiguous readout of the chromatin location, it is important to separate the fluorescent and CID fusion proteins. This experimental scheme is based on exogenous factors that have no specific binding partners in the native genome, so the interactions should be established with high specificity at the target sites. The interaction should have considerable strength due to a large number of FKBP12-FRB contacts over the LacO and TetO repeats (256X and 192X). The addition of rapamycin allows the interaction to be manipulated dynamically.

For this assay to work, it is essential to keep the concentrations of the LacI-FKBP12 and TetR-FRB at low levels: freely diffusing proteins dimerize with the chromatin-bound counterparts and therefore interfere with the interaction between the two targeted loci. Based on our previous study^26^, we constructed CICI strains with *REV1* promoter driving the LacI-FKBP12 and TetR-FRB fusion proteins, which can maintain high occupancy of LacO/TetO arrays with low free protein concentrations (Methods). For comparison, we also generated control strains containing LacI and TetR instead of LacI-FKBP12 and TetR-FRB, so that no induced interactions can form. The CICI and control strains, as well as strains with LacO/TetO arrays alone, have similar growth rates as the rapamycin-resistant background strain (Supplementary Fig. 1A). They also contain the expected number of fluorescent chromatin dots (Supplementary Fig. 1B). We, therefore, conclude that, in our strains, these genetic components have a little direct impact on cell cycle progression and genome stability.

As a proof of principle, we first tested CICI with a loci pair that is known to have a high tendency to interact. Chromosomal interactions in yeast are strongly constrained by the Rabl configuration where centromeres cluster, and the neighboring chromosome arms run closely in parallel^27–29^. As a result, Hi-C experiments showed that loci with equal distances to centromeres have higher contact frequencies^28^. We, therefore, inserted LacO and TetO arrays into the left and right arm of Chr XV, both ~173 kb away from the centromere (pair 1) (Fig. 1B). We incubated these cells with or without rapamycin for 1 h, imaged under a fluorescent microscope, and analyzed the distance between the LacO and TetO dots in individual cells (Methods). The LacO and TetO dots become closer in the presence of rapamycin (Fig. 1C, D). In contrast, LacO-TetO distances in the control strain that lacks the CID proteins do not change with rapamycin (Fig. 1C, D), showing that the LacO-TetO co-localization is not a generic reaction to rapamycin, but represents true CICI events. To measure the rate of CICI formation, we imaged cells at different time points after adding rapamycin. The co-localization probability rapidly increases from ~40% to more than 70% within 20 min in the CICI strain, but remains constant in the control strain (Fig. 1E).

### CICI formation at loci with different Hi-C contact frequencies

**Fig. 2:**
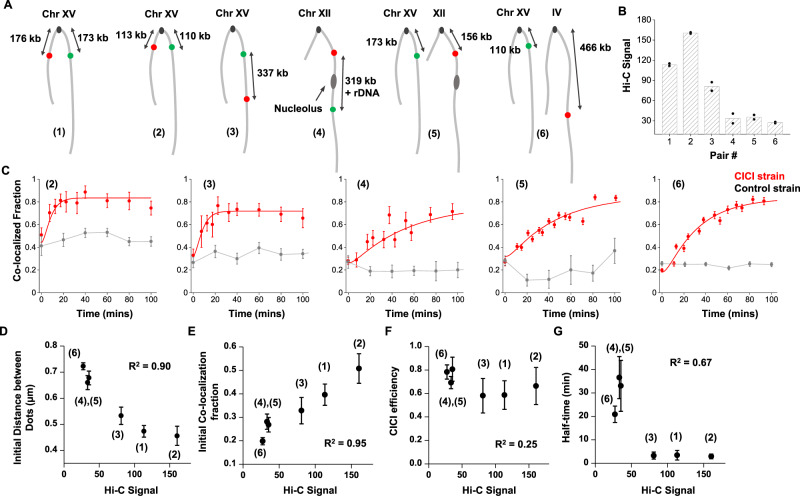
CICI formation at loci with different Hi-C contact frequencies. **A** Six loci pairs were chosen for the CICI test. **B** Hi-C signals of the six pairs of loci based on published datasets (see Methods and Supplementary Data 1 for details). Data shown are the average of two biological replicas (individual data points are also shown). **C** The dynamics of CICI formation for loci pairs 2–6 (from left to right). The total numbers of data points were 387 (CICI) and 764 (control) for (2), 430 and 713 for (3), 724 and 338 for (4), 5376 and 228 for (5), and 4830 and 3156 for (6). The smooth curves are exponential fitting of the data (Methods). Error bars represent standard error (same for **D**–**G**). **D**–**G** The correlation between Hi-C signal with the initial distance between the dots (**D**), initial co-localization probability (**E**), CICI efficiency (**F**), and the half-time of CICI formation (**G**). The initial distance and co-localization probability were derived from the data shown in Fig. 1E and **C** at *t* = 0 time points, and CICI efficiency and the half-time were calculated from the fitting parameters. Points represent loci pairs 6, 4, 5, 3, 1, and 2 from left to the right. The corresponding coefficients of determination *R*^2^ are shown in the panels. Source data for **B**–**G** are provided as a Source Data file.

Given that CICI can be successfully established for the loci above, we next generated five more strains with different LacO/TetO insertion sites (pairs 2–6 in Fig. 2A). These loci pairs were chosen strategically to span a broad range of contact frequencies based on published Hi-C data^27,28^ (Fig. 2B) (Methods). In comparison to pair 1, the Hi-C signal is higher for pair 2 (symmetric to centromere with shorter linear distance) lower for pair 3 (similar linear distance but on the same chromosome arm), and even lower for pairs 4–6. For pair 4, LacO and TetO were placed on the two sides of rDNA, which separates Chr XII into two isolated domains. According to previous Hi-C measurements, the two domains have very low contact frequencies^28^. For pairs 5 and 6, LacO and TetO were inserted into two different chromosomes, and the frequencies of “inter-chromosomal” interactions are generally lower than those of “intra-chromosomal” interactions. Upon induction with rapamycin, we observed CICI formation for all loci pairs despite the fact that some of them are well-separated in the absence of rapamycin (Fig. 2C and Supplementary Fig. 2). We also used 3C to confirm the contact formation for pairs 3 and 5 (Methods). As expected, the 3C PCR product is only visible in the CICI strain when induced with rapamycin (Supplementary Fig. 3).

To quantify the relation between CICI formation and Hi-C contact frequency, we derived the initial mean distance between dots, initial co-localization probability, CICI efficiency (net co-localization gains at steady state), and *t*_1/2_ of CICI formation for each loci pair using the data in Fig. 2C (Methods), and plotted these variables against their Hi-C signals. In the absence of rapamycin, the Hi-C signals show a strong negative correlation with the dot distance, and a strong positive correlation with the initial co-localization probability (*R*^2^ > 0.9; Fig. 2D, E). Such correlations are expected because Hi-C measures the probability for two loci to fall within a ligatable distance, which is largely determined by the average distance in-between. In striking contrast, the Hi-C signals have less correlation with the CICI efficiency (*R*^2^ = 0.25; Fig. 2F), i.e., for all the CICI pairs generated, interactions can form in 60–80% of cells regardless of their initial 3D conformation. The rate of contact formation, however, depends on the initial conformation: for pairs 4–6 with low Hi-C contact frequencies, CICI formation is almost one order of magnitude slower than the other three pairs (Fig. 2G). Overall, these data reflect the kinetic nature of the Hi-C signals: two loci with low contact frequency can still form interactions, but it takes longer for them to encounter.

### CICI formation in real time

**Fig. 3:**
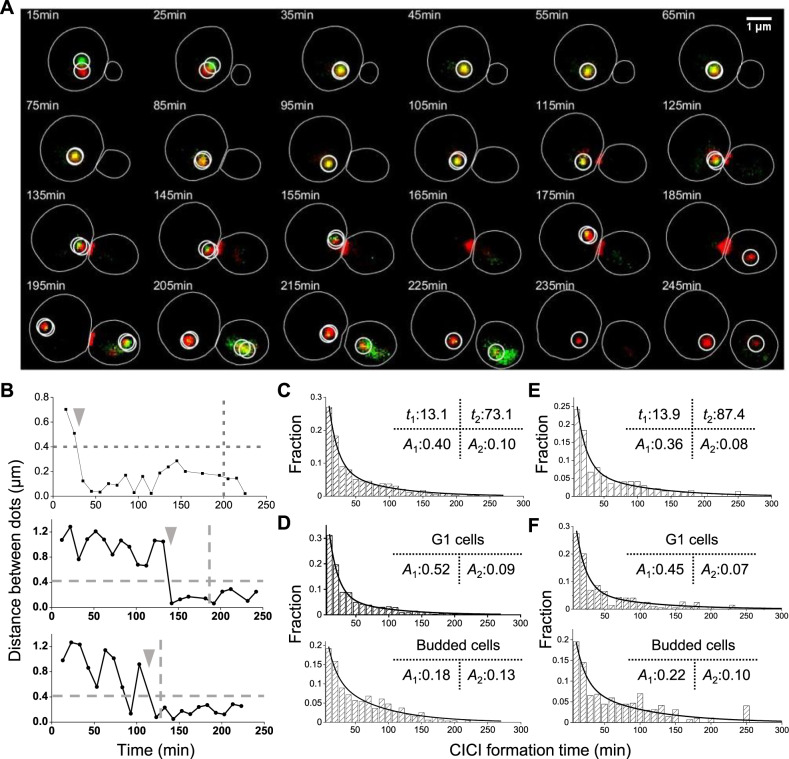
CICI formation in real time. **A** Example time-lapse images of CICI strain (pair 5). The chromatin dots at the LacO and TetO arrays are circled. The budneck is labeled with Myo1-mCherry. **B** Distance between LacO and TetO arrays as a function of time in three cells. The upper panel is derived from the mother cell in (**A**). The horizontal line represents the co-localization threshold (0.4 µm), and the vertical lines show the time of cell division. The gray arrows point to the CICI formation time in these three single cells. **C** Histogram of CICI formation time for all cells (LacO and TetO pair 5, *N* = 773). The histogram is fitted with double exponential curves, and the time constants and amplitudes of the two phases (*t*_1_, *t*_2_, *A*_1_, and *A*_2_) are shown. **D** Histogram of CICI formation time for cells divided into two different cell cycle stages (*N* = 480 and 255 for G1 and budded cells, respectively). The double exponential fit has the same *t*_1_ and *t*_2_, but different *A*_1_ and *A*_2_. **E**, **F** Same as **C** and **D**, except that LacO and TetO arrays are placed on the two sides of the nucleolus (pair 4). *N* = 378, 220, 135 for all cells, G1 cells and budded cells, respectively. Source data for **B**–**F** are provided as a Source Data file.

The time course in Fig. 2C measures the average CICI formation time among the cell population. To measure this time at the single-cell level, we carried out time-lapse imaging to directly visualize CICI formation between loci pair 5 (the inter-chromosomal pair) in real time (Methods). A typical movie clip is shown in Fig. 3A, and the distance between the dots as a function of time in three cells are shown in Fig. 3B. In these examples, the two dots are initially separated before showing continuous co-localization, clearly demonstrating the CICI formation processes. The time-lapse measurements can recapitulate the static measurements of co-localization probability in Fig. 2C (Supplementary Fig. 4). The strain used here also contains a cell-cycle marker, Myo1-mCherry, which forms a ring at the budneck that disappears upon cell division^30^. We determined the cell division times based on the Myo1 signal and marked these times as a vertical bar in Fig. 3B. For the three cells shown here, CICI persisted through the cell divisions.

We extracted the CICI formation time from each single cell (arrows in Fig. 3B) and plotted the histogram in Fig. 3C (Methods). The distribution fits better to a double than a single exponential curve (AIC test shown in Supplementary Fig. 5). The time constants for the two phases are 13 and 73 min, respectively, indicating that CICI forms much faster in a fraction of cells. Interestingly, such a rate is affected by the cell cycle: 54% of G1 cells follow the faster CICI formation dynamics, while in budded ones, this number is only 20% (Fig. 3D). We repeated the same measurements with CICI pair 4 and found the same trend (Fig. 3E, F and Supplementary Fig. 5). The biphasic behavior is less prominent for pair 6, but the CICI formation time is still slightly longer in budded than unbudded cells (Supplementary Fig. 5C, G). These differences in CICI formation rate is likely related to the constrained motion of chromosome after DNA replication (see Discussion).

### CICI is disrupted shortly before cell division in a fraction of cells

**Fig. 4:**
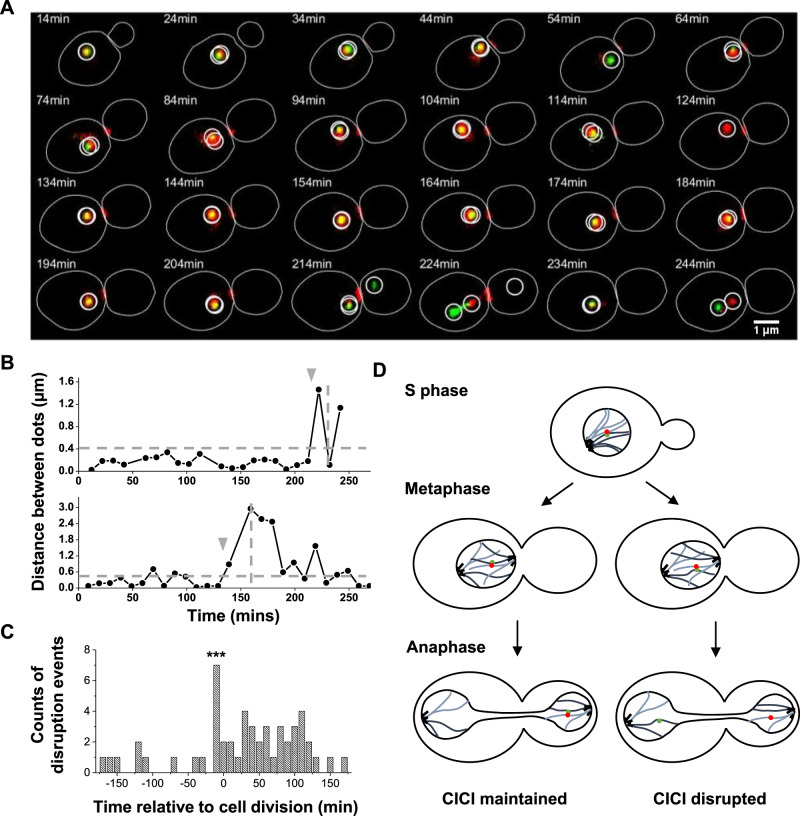
CICI is disrupted shortly before cell division in a fraction of cells. **A** Example time-lapse images of CICI strain (pair 5) that show CICI disruption near the end of the cell cycle. **B** Distance between LacO and TetO arrays as a function of time in two cells. The upper panel is derived from the mother cell in (**A**). The horizontal line represents the co-localization threshold (0.4 µm), and the vertical lines show the time of cell division. The gray arrows point to the time of CICI disruption. **C** Histogram of the time of CICI disruption relative to the end of the cell cycle (total CICI disruption events *N* = 36). The seven disruption events near the end of the cell cycle are statistically significant (one-sided Student’s *t*-test, *P* value < 0.001). **D** Model of CICI disruption during anaphase. For clarity, only one of the two CICI pairs is shown in these cells. Source data for **B** and **C** are provided as a Source Data file.

Time-lapse imaging also revealed that CICI can be disrupted. In Fig. 4A, for example, the CICI pair 5 remained co-localized for more than 200 min before they were separated. Based on the Myo1 marker, this cell divided between 224 and 234 min, so the disruption happened shortly before the cell division. From the distance vs time curves (examples shown in Fig. 4B), we determined the disruption times relative to the cell divisions (Methods). The histogram of the disruption time shows a distinct peak around –10 min (Fig. 4C), indicating that CICI disruption tends to occur during the anaphase. Among the cells that we can unambiguously determine the fate of CICI across cell division, there are 47 intact and 11 disrupted. These observations lead to the model in Fig. 4D: after DNA replication, the cells contain two copies of LacO and TetO arrays, which will be split into mother and daughter cells during mitosis. If CICI is formed between two arrays that will end up in the same cell, CICI can be maintained through cell division. In contrast, if CICI is formed between two arrays that are going into different cells, CICI has to be disrupted to resolve this topological conflict.

We also observed some disruption events at other cell cycle stages (example shown in Supplementary Fig. 6A, B), and these events are rather uniformly distributed across the cell cycle (Fig. 4C). We initially suspected that DNA replication may be responsible for some of these disruptions, as the passage of DNA polymerase likely requires transient dissociation of the bound LacI and TetR proteins. However, when we formed CICI in G1-arrested cells and released them into S phase, we did not observe a synchronized loss of CICI (Supplementary Fig. 6C). The mechanism of the CICI disruption during the interphase is still unclear. It is possible that the movement of chromosomes inside the nuclei can occasionally generate enough force to separate the CICI contacts. Regardless of the mechanism, the fact that CICI can be disrupted explains why the steady-state CICI levels do not reach 100% after long-term rapamycin incubation (Fig. 2C).

### CICI affects the donor preference in homology-directed DNA repair

The ultimate goal of developing CICI is to perturb chromosome conformation and probe its biological consequences. Here, we performed two functional tests of CICI. First, we examined the effect of CICI on DNA homology-directed repair. Long-range interactions between homologous sequences should play an essential role in this process as the repair template needs to be in close vicinity of the DNA lesion^31,32^. Mating-type switching is a well-characterized model for homologous repair in budding yeast^33,34^. The switching is initiated by the *HO*-mediated double-stranded break at the *MAT* locus, which is then repaired by *HMLα* or *HMRa* located on the opposite ends of Chr III. The donor preference (*MATa* is mostly repaired by *HMLα*, and *MATα* by *HMRa*) is thought to result from differential configurations of Chr III in a and α cells. However, *MAT-HML* and *MAT-HMR* contact frequencies only have mild differences in cycling a and α cells^27^. Previous studies have used tetrameric LacI to alter the Chr III conformations^16^ or membrane-tethered LacI to constrain the motion of *HML*^35^, and observed some changes in the donor preference. However, how efficiently these methods perturb the proximity of *MAT-HML* and *MAT-HMR* is not clear. Here, we directly induced interaction between *MAT* and *HMR* in *MATa* (a mating type) cells to see if we could rewire the donor preference.

**Fig. 5:**
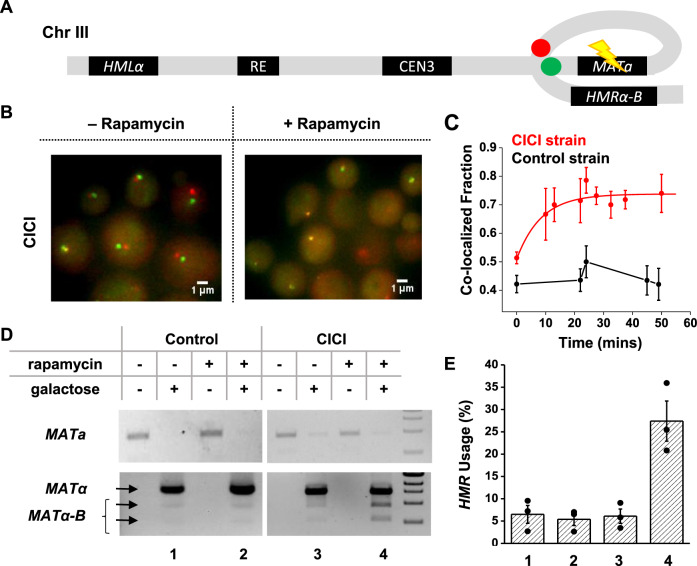
CICI affects the donor preference in homology-directed DNA repair. **A** Engineered proximity between *MATa* and *HMRα-B* by CICI. Green and Red dots: LacO and TetO arrays; Flash symbol: HO digestion. **B** Visualization of the *MATa* and *HMRα-B* loci ± rapamycin. **C** Fraction of co-localization as a function of rapamycin incubation time in the CICI (red) or the control strain (black) used in the mating-type switching assay. The total numbers of data points were 226 for the CICI strain and 272 for the control. Error bars represent standard error in the fraction. **D** Quantitative analysis of *HMR* usage in yeast mating-type switching. Top gel: PCR of the *MATa* sequence, which decreases after the *HO* induction (representing the switching from a to α or α-B). Bottom gel: PCR of the *MATα* sequence followed by BamHI digestion. Undigested (top arrow)/digested bands (bottom two arrows) represent the usage of *HML* (α) and *HMR* (α-B). 1 kb DNA ladder is shown on the right. **E** The fraction of *HMR* usage for the four conditions in **D**, which was calculated as the sum intensity of the lower two bands divided by the sum intensity of all three bands. Data are presented as mean values ± SEM from three biological repeats. Source data for **C**–**E** are provided as a Source Data file.

We constructed a *MATa* yeast strain containing the CICI fusion proteins with LacO and TetO ~5 kb downstream the *MAT* and *HMR* loci (Fig. 5A), as well as a control strain lacking FKBP12 and FRB. We confirmed that CICI can form in this strain with a ~50% efficiency, but not in the control (Fig. 5B, C). This strain also has *GAL10pr-HO* and *HMRα-B* (*HMRa* replaced by *HMRα* with an engineered BamHI cutting site^35^), allowing us to induce the *MAT* repair with galactose and probe the relative use of the *HML* and *HMR* based on BamHI digestion (Methods). We grew these strains to log-phase in raffinose, added rapamycin for 1 h to establish CICI, and induced the *HO* expression with galactose for 1 h. We then added glucose to repress *HO* for 1 h, allowing repairs to take place, before extracting the genomic DNA and genotyping the mating locus (Methods). The presence of rapamycin increases the usage of *HMRα-B* in the CICI strain from 6 to 27%, but shows no effect in the control strain (Fig. 5D, E). These data demonstrate that the engineered chromosomal interaction in Fig. 3A can significantly override the endogenous mechanism of donor selection during mating-type switching.

### CICI has no effect on replication timing

We next used CICI to perturb the chromosome conformation near replication origins and probed the consequence in replication timing. Replication origins encoded by autonomously replicating sequences (ARSs) have different initiation timings during the S phase. Interestingly, replication timing is often spatially organized along chromosomes, e.g., origins close to each other on the genome have more similar timings. In particular, origins near centromeres tend to fire earlier than those near telomeres^36–42^. Early and late origins are not intrinsically encoded by their DNA sequences^37^; instead, replication timing can be altered by relocating the ARSs to new genomic loci^43^. Recent studies found that early origins cluster in the 3D space^28,44–47^, and yeast chromosomes are organized into topological associating domains (TADs) that are strongly correlated with replication timing^48^. Despite these correlations, the causal relation between chromosome conformation and replication timing remains unclear.

**Fig. 6:**
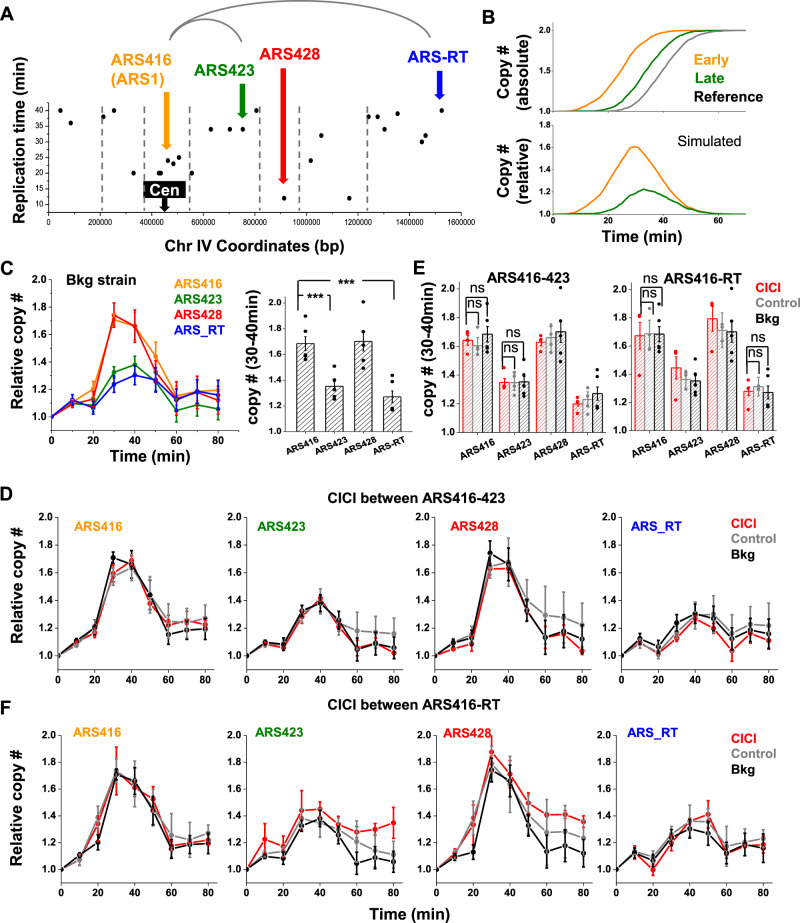
CICI has no effect on replication timing. **A** The replication timing of different ARS sequences (data from Raghuraman et al.^37^). The vertical lines represent TAD boundaries^48^. We choose four ARSs in four different TADs, ARS416, ARS423, ARS428, and ARS-RT for further testing. **B** Expected copy number as a function of time for different ARS sequences, as well as the relative copy number normalized by a control region with late replication. **C** The relative copy number as a function of time of the ARS sequences highlighted in **A**. Five biological repeats were included. The bar diagram on the right shows the average copy number of each ARS between 30 and 40 min after G1 release. Error bars represent standard error (same for panels below). The differences between ARS416 and ARS423/ARS-RT are statistically significant (one-sided Student’s *t*-test, *P* values < 0.001). **D** Consequence of CICI between ARS416 and ARS423 on replication timing (four biological repeats for each of CICI and control). The four panels show the relative copy number of the four ARSs in the CICI strain (LacO and TetO inserted near ARS416 and ARS423), control strain (same LacO and TetO arrays, but LacI and TetR are not conjugated to FKBP12 and FRB), and background strain (no CICI components). All the measurements were carried out in the presence of rapamycin. **E** Average ARS copy number between 30 and 40 min derived from data in **D** and **F**. No significant difference in replication timing was detected in CICI condition (two-sided Student’s *t*-test). **F** Same as in **D** except the CICI is between ARS416 and ARS-RT (TetO inserted near ARS-RT in the CICI and control strains, three biological repeats for each of CICI and control). Source data for **B**–**F** are provided as a Source Data file.

To test the role of chromosome conformation in replication timing, we used CICI to bring two distant ARSs into physical proximity and examined the effect on replication timing. We selected four ARSs that locate in different TADs on Chr IV^48^, including two early-firing ARSs, ARS416 (aka ARS1) and ARS428, and two late ones, ARS423 and ARS-RT (RT represents the right telomere) (Fig. 6A). All four origins have well-defined ARS consensus sequences situated in broad nucleosome-depleted regions^49^ (Supplementary Fig. 7A, B), therefore, the differences in their timings are unlikely due to motif strength or chromatin environment. To measure the replication timing of these origins, we arrested cells in G1 by α-factor, released into S phase, purified genomic DNA at different time points, and used qPCR to measure the DNA copy number near the ARSs. These copy numbers were normalized by a control region that is far from any ARSs and thus exhibits late replication (Methods). Figure 6B shows the expected relative DNA dose as a function of time, where the early origin shows a strong pulse that peaks early, and the late one is flatter. In the background strain (strain that is rapamycin resistant but has no CICI components), the measurements near the four ARSs are consistent with this expectation (Fig. 6C). For easier comparison, we also plotted the average DNA copy number between 30 and 40 min after G1 release, where higher values represent earlier ARSs.

We engineered LacO and TetO arrays near ARS416 (early) and ARS423 (late). Because the bound LacI and TetR may form barriers for replication fork^50^, the arrays were inserted ~2 kb away from the ARSs either downstream or on the opposite side of the qPCR probes (Supplementary Fig. 7C). After α-factor and rapamycin treatment, CICI can be established in 30~45% of G1-arrested cells after 1 h (note that G1-arrested cells tend to have larger sizes, which is likely to be responsible for the reduced CICI efficiency) (Supplementary Fig. 7D). We then released the cells into S phase to perform the replication measurement (Fig. 6D). For comparison, we carried out the same measurement on the background and control strain (containing all CICI components except for FKBP12 and FRB). The replication timings of ARS416 and ARS423 are comparable in all three strains (*p* value = 0.31 and 0.19 for ARS416 between CICI and control or background, *p* value = 0.97 and 0.86 for ARS423) (Fig. 6E), indicating that CICI between ARS416 and ARS423 has no significant impact on their replication timing. To further test this conclusion, we generated CICI between ARS416 (early) and ARS-RT (late) (Fig. 6F). Again, the replication timings of these origins remain intact (*p* value = 0.87 and 0.86 for ARS416 between CICI and control or background, *p* value = 0.51 and 0.76 for ARS-RT) (Fig. 6E). These observations indicate that, at least for some ARSs, the 3D localization does not directly determine their replication timing (see Discussion).

## Discussion

In this study, we developed a synthetic biology method, CICI, to induce chromosomal interactions between targeted loci. Using this method, intra- and inter-chromosomal interactions can be engineered and dynamically controlled through a diffusive chemical. Some of the loci pairs tested here have very low Hi-C contact frequencies and on average, large physical separations (Fig. 2). Nevertheless, CICI forms among all of these loci pairs in a large fraction of cells, reflecting considerable flexibility in 3D chromosomal conformation. Given that CICI can be established in some extreme cases (e.g., across the two sides of nucleolus), we suspect that most, if not all, intra-chromosomal loci pairs can make contacts at a biologically relevant time scale, e.g., within a cell cycle. The fact that interactions can be induced between loci with low Hi-C signals is consistent with analyses of chromosome conformation in higher eukaryotes, which revealed that loci pairs in different TADs can be in physical proximity in a fraction of cells^51^. Given our observation, we suspect that intra-TAD interactions have kinetic advantages over inter-TAD ones. However, interactions can eventually form when they are induced across TAD boundaries, especially in the absence of competition.

CICI formation time reflects the rate of chromosome encounter. Since many nuclear processes, such as DNA repair or enhancer-promoter looping, involve interactions among distant chromosomal loci, such encounter time is physiologically important. In principle, the encounter time can be evaluated by tracking two loci in live cells and measuring how long it takes for the two loci to move within a distance threshold. However, due to limited spatial resolution, we cannot be certain if the two loci within the threshold form functional interactions. In contrast, CICI causes prolonged co-localization of the loci over time, allowing us to catch the encounter events unambiguously. In principle, CICI formation dynamics should be dictated by the physical distance and the diffusion properties of the two loci. The slow CICI formation time in budded cells is consistent with the previous observation that the motion of chromosome loci is more vigorous in G1 than S phase^52^. The tethering of the sister chromatids is likely to be responsible for such slow motion. Indeed, cleavage of cohesin was shown to enhance the movement of yeast chromosome in S phase^53^.

Strong interaction between chromosomes that need to be separated during mitosis can potentially be disastrous. We therefore originally expected to see wide-spread cell cycle arrest and death after CICI induction. Surprisingly, the doubling time of CICI strains are very similar with or without rapamycin (Supplementary Fig. 1), indicating that CICI does not pose a major barrier for cell cycle progression. This is consistent with our observation that CICI can be disrupted, especially during anaphase. These results imply that the force generated by the mitotic spindle is sufficient to separate FKBP12 with FRB or LacI/TetR with DNA. Note that it is unlikely the CICI disruption is caused by chromosome compaction and CICI component eviction, because (1) LacI-FKBP12/TetR-FRB binding should be similar to that of LacI-GFP and TetR-mCherry, which, based on fluorescence measurement, are not evicted from the chromatin in the mitotic cells, and (2) disruption tends to happen right before cytokinesis, but chromosome condensation happens at an earlier time point. Interestingly, events that require dissociation of DNA-bound proteins, such as DNA replication, do not seem to have a major disruptive effect on CICI (Supplementary Fig. 6C). We suspect that this is because the passing DNA polymerases only disrupt a fraction of the bound LacI and TetR at a time. The two loci continue to be tethered through the remaining bound proteins, and the dissociated LacI and TetR can rapidly rebind and reestablish CICI contacts.

We carried out two functional tests by perturbing the chromosome conformation with CICI and probing the consequences in DNA repair and replication timing. These studies allow us to go beyond correlation and directly test the causal relation of chromosome topology in these processes. For DNA repair at the *MAT* locus in *MAT*a cells, the preference of using *HML* may be due to its physical proximity and/or higher efficiency of *HML* recombination. Our results demonstrate that physical proximity indeed plays a role in donor preference. Previous Hi-C measurement showed that *MAT* locus and *HMR* can make contacts even in *MAT*a cells^27^. Our data indicate that the proximity of the two loci can be further enhanced, which makes *HMR* more competitive in recombination. Why is the *HMR* usage only increased to 27% with CICI? One reason is that CICI only induces co-localization in ~50% of cells, and therefore about half of the cells will likely follow the default donor preference. In addition, even when *HMR* is brought into proximity with *MATa*, it still needs to engage in kinetic competition with *HML*. If we induce the looping between *HMR* and *MATa* while restraining the motion of *HML* (like in Avşaroğlu et al.^35^), the *HMR* usage is likely to be further enhanced.

Replication timing in many eukaryotic species is known to correlate with 3D genome organization such as A/B compartments and TADs^1,48^, and it is intriguing that 3D organization may play a deterministic role in replication timing. For example, it was proposed that replication timing is established during G1 due to the differential loading of a rate-limiting factor, Cdc45p^54–56^, and the genome can be organized into early- and late-replicating TADs that differ in the local Cdc45p concentration^36,44,48^. This type of model would predict that origins that are physically proximal, including the ones that are forced into proximity, share a similar micro-environment and therefore should have similar replication timing. However, our data show that forced co-localization of early and late ARSs does not impact their replication timing. This result indicates that, at least for some ARSs in budding yeast, replication timing and 3D localization can be decoupled. The underlying mechanism requires further investigation, but our data are more consistent with replication timing being determined by locally bound factors and/or chromatin states rather than global nuclear genome organization.

In summary, we developed CICI as a generic method to engineer specific 3D chromosome interactions, and applied this tool to study chromosome dynamics and 3D genome function. Because the two techniques that CICI is based on, CID and FROS, have both been implemented in multicellular eukaryotic cells^57,58^, we expect that CICI can be constructed in these model organisms and have broad applications.

## Methods

### Plasmid and strain construction

Standard methods were used to construct the strains and plasmids (see Supplementary Table 1 for plasmid and strain information). All the strains used in this study were derived from a background strain carrying *tor1* and *fpr* mutations for rapamycin resistance. These rapamycin-resistant strains grow and divide slowly with a doubling time of ~2.5 h at 30 °C. The insertion loci were selected to be within intergenic regions between convergent gene pairs to have minimal effects on the endogenous gene expression (see Supplementary Table 1 for insertion index information). We constructed the CICI strain by integrating the plasmid containing LacI-FKBP12 and TetR-FRB driven by *REV1pr* into the *ADE2* locus. For the control strain, we integrated the plasmid containing LacI and TetR driven by *REV1pr*. We then integrated a plasmid containing *REV1pr*-LacI-GFP and *REV1pr*-TetR-mCherry into the *HIS3* locus to allow fluorescence detection of the LacO and TetO arrays. Since these arrays contain repetitive sequences that are difficult for molecular cloning, we used a two-step integration method developed by the Gasser lab^59^. We first integrated a *URA3* selection marker into the target site, and then replaced *URA3* by LacO array through FOA selection. The same protocol was repeated subsequently for the TetO integration, but with a different sequence flanking the *URA3* marker. After every step of strain construction, yeast colony PCR was performed to verify the integration site. To keep track of the cell division, we replaced the endogenous *MYO1* by *MYO1-mCherry* flanked by *KanMX* marker. The budneck has the same fluorescence as the TetO arrays. However, the budneck is located between mother and daughter cells and is thus distinguishable from TetO arrays.

For strains used in the mating-type switching assay, we integrated the LacO/TetO arrays into the intergenic regions ~5 kb downstream the *MAT* and *HMR* loci with the same method, so that the insertion would not disrupt the mating loci, and the homologous recombination could happen in the right orientation (see Supplementary Table 1 for insertion index information). We also mutated the *HMRa* sequence to *HMRα-B*, which can be differentiated from *HMLα* by BamHI digestion. This manipulation is done through mating with a strain containing *HMRα-B* (generously provided by Dr Haber) and tetrad dissection. In addition, we inserted a *GAL10pr-HO* into the *GAL10* locus to express *HO* in the strain. For the replication assay, we integrated the LacO/TetO arrays into the intergenic regions ~2 kb either downstream or upstream of the selected ARSs (Supplementary Fig. 7C).

### Hi-C data collection

The Hi-C data shown in Fig. 2 is extracted from Belton et al.^27^ (GSM1905067 and GSM1905068). In their datasets, they divided the whole genome into 10 kb bins, and calculated the normalized contact frequency for each pair of bins. Since both the LacO and TetO array have a length close to 10 kb, their intrinsic contact frequency should be reasonably mimicked by the contact frequency of the 10 kb bins near their insertion sites. The original Hi-C data near our CICI pairs are shown in Supplementary Data 1. We also collected Hi-C data from two more datasets, Lazar-Stefanita et al. and Schalbetter et al.^60,61^. Both of these measurements were carried out in a synchronized cell population. These datasets, regardless of the cell cycle stages, are well correlated with Belton et al. (Supplementary Fig. 8), confirming the validity of these contact frequency values.

### CICI image acquisition

For steady-state images, we grew the log-phase cells in glucose, added rapamycin (Sigma catalog # 53123-88-9) to a final concentration of 1 ng/ul or an equal volume of DMSO, and incubated for 1 h. We then transferred the cells onto an agarose pad containing the same concentration of rapamycin/DMSO and further incubated for ~10 min for the cells to adjust to the solid medium. The agarose pad was then laid onto a glass coverslip and imaged under a Leica fluorescent microscope (Leica DMI6000 with Hamamatsu ORCA-R2 C10600 camera) in phase, GFP, and mCherry channels^30^. Nine z-stacks were taken with an exposure time of 0.2 s and light intensity of 100% (SOLA SE light source) for both GFP and mCherry channels. For CICI time course, we directly placed log-phase cells onto an agarose pad with 1 ng/ul of rapamycin and started imaging immediately. The incubation time for each set of z-stack images was calculated as the duration between rapamycin addition and time of image acquisition. For each strain, we carried out at least two independent time-course measurements, and we did not notice any significant batch-to-batch differences. For each time point in each measurement, we took seven images (each image contains tens of cells) for CICI or control strains and pooled the data from all the cells together in our final plot.

For single-cell tracking experiments, we first inoculated cells in synthetic complete media with glucose (SCD) to log-phase without rapamycin. At *t* = 0 min, we induced CICI by adding rapamycin to a final concentration of 1 ng/ul. Then we transferred cells to an agarose pad containing the same concentration of rapamycin, and started time-lapse imaging using a 100× objective^30^. To mitigate photodamage, we reduced the fluorescence excitation to 0.1~0.15 s, used seven z stacks with 0.6 µm spacing, and chose 10-min interval between frames. The time of cell division was determined based on the disappearance of *MYO1-mCherry* cell cycle marker.

For G1 arrest experiments, we first arrested log-phase cells in G1 by adding 50 nM α-factor (Zymo research catalog # Y1001), and then incubated these cells for 1.5 h with rapamycin to induce CICI. To release cells from G1, we washed the cells twice in media containing 0.1 mg/ml pronase and 1 ng/ul rapamycin. For the control cells, we washed the cell twice in media containing 50 nM α-factor and 1 ng/ul rapamycin to keep the cells G1-arrested. Then we transferred both G1-arrested and released cells onto two separate agarose pads on the same glass slide to start time-lapse imaging. Both pads contain 1 ng/ul rapamycin, and the pad for G1-arrested cells also contains 50 nM α-factor.

### Image analysis

We developed Matlab software to analyze the z-stack fluorescent images^26^. After using the phase image to annotate the cell boundaries, we first generated a composite image where each pixel within the cell boundaries takes on the maximum intensity of this pixel among the z-stack. We then detected the locations of the green and red chromosome dots with intensities above a threshold. We recorded the *x* and *y* positions of the center of the dots and then calculated the distance in-between. For most cells, one green dot and one red dot are detected by the program (Supplementary Fig. 1). For cells containing more than one green or red dot, which might be due to DNA replication, we calculated the pairwise distances between each green and red dot and recorded the minimum distance. Green and red dots within 0.4 µm were considered co-localized. The raw distance measurements for CICI and control strains pairs 1–6 are shown in Supplementary Data 2.

For single-cell tracking imaging analysis, we calculated the minimum distance between red and green dots in each cell at each frame. Since co-localized dots do not necessarily represent real contact between the two loci, we required the co-localization to last at least three continuous frames to be annotated as CICI formation. After CICI formation, the contact is considered to be disrupted if the co-localization is lost at least twice within four frames, or if the distance is larger than 0.6 µm. Due to photobleaching, photodamaging, and other technical issues, the signal quality decreases over time. We focused on traces that maintain clear dot signals in more than 80% of the frames.

### CICI formation data analysis

The observed CICI formation time should include both the time for rapamycin to diffuse into the nucleus and the time for the two chromosomal loci to find each other. Assuming these are consecutive and independent Poisson processes, each of them following an exponential probability density function, we fitted the CICI formation curves with the following equation (the convolution of the two Poisson distributions):

1 $${P}_{{{{{{\rm{co-localization}}}}}}}={a}_{1}+\frac{{a}_{2}}{{k}_{2}-{k}_{1}}\times \left(\frac{1}{{k}_{1}}(1-\exp (-{k}_{1}t))-\frac{1}{{k}_{2}}(1-\exp (-{k}_{2}t))\right)$$

where *k*_1_ and *k*_2_ represent the rate of the two processes, and *a*_1_ and *a*_2_ determine the initial and final co-localization probability. We fixed the *k*_2_ (the rate for rapamycin to enter the nucleus) to 0.2 min^–1^ for all the CICI strains. *t*_1/2_ was calculated as ln2/*k*_1_. CICI efficiency is defined as the net gain of co-localization probability after reaching steady-state, which is calculated as follows:

2 $${{{{{\rm{CICI}}}}}}\; {{{{{\rm{efficiency}}}}}}=({P}_{{{{{{{\rm{final}}}}}}}}-{P}_{{{{{{{\rm{initial}}}}}}}})/(1-{P}_{{{{{{{\rm{initial}}}}}}}})$$

### 3C assay

We adapted previously published 3C methods^62,63^. In all, 2 × 10^9^ cells in 200 ml SCD were fixed in 3% formaldehyde (Sigma) for 20 min at 25 °C, and then quenched with 35.28 ml of 2M glycine for 20 min at room temperature. Cells were recovered by centrifugation and washed with the same medium. The cell pellet was resuspended in 1 ml TBS, 1% Triton-X and 1X protease inhibitor cocktail (Thermo Scientific). Cell lysis was obtained by adding 500 μl acid-washed beads (Sigma) and vortexing for 5 min, 5 times with 2-min break on ice. The chromatin was recovered through centrifugation, washed with 1 ml TBS, resuspended in 500 ul 10 mM Tris buffer and digested with DpnII (NEB) overnight at 37 °C. The digested DNA fragments were ligated by T4 DNA ligase (Thermo Scientific) for 4 h at 16 °C. Crosslink was reversed by incubation with proteinase K at 65 °C overnight. DNA was purified through a phenol/chloroform extraction. RNA was removed through an RNase A treatment, and DNA was purified again by DNA clean and concentrator-5 Kit (Zymo). Primers were designed for the regions of interest near DpnII cutting sites (Supplementary Table 2), and PCR reactions were performed for testing interactions between these regions.

### *MAT* repair assay

We adapted the protocol developed by the Haber lab^64^. Single colonies were grown overnight in 5 mL YEPD (YEP+ 2% Glucose, for no *HO* control) or YEPR (YEP+ 3% Raffinose, for HO induction) media with a yeast hormone “a factor” (10 ng/ul) at 30 °C to log phase. We noticed a high background switching frequency from a cell to α cell in YEPD where *GAL10pr*-*HO* was not induced, and 80 uM of “a-factor” (Zymo Research catalog # Y1004-500) was added to inhibit the growth of switched α cells before *HO* induction. We added 1 ng/ul rapamycin to these cultures to induce CICI formation between *MAT* and *HMR* for 1 h (DMSO added as the control). We washed away the a-factor, added back rapamycin (or DMSO), and induced the expression of *GAL10pr*-*HO* for 1 h with YEPG (YEP+ 3% Galactose). Glucose was then added to a final concentration of 2% to stop the *HO* expression, and cells were incubated for an additional 1 h to allow DNA repair. We next extracted genomic DNA from these samples and tested the genotype by PCR. To differentiate *MATa* vs. *MATα* (total switching), we used a forward primer that is immediately adjacent to the *MAT* locus and a reverse primer specific to either a or α sequence. To test the usage of *HML* vs. *HMR* among the switched population, we digested the *MATα* PCR products with BamHI and quantified the digested fraction using ImageJ. The undigested band (*MATα*) represents *the HML* usage, and two digested bands (*MATα-B*) represent the *HMR* usage. The total usage of *HMR* is calculated as the *MATα-B* band intensity divided by the total intensity of *MATa* and *MATα-B*.

### DNA replication timing measurement

We adopted the method from Batrakou et al.^65^. We grew cells in SCD-hlta media overnight to OD_660_ around 0.2, and then added α-factor to a final concentration of 50 nM and rapamycin to a final concentration of 1 ng/ul. We harvested the G1-arrested cells after 3 h and washed the cells with cold SCD-hlta media containing 0.1 mg/ml pronase and 1 ng/ul rapamycin to remove the α-factor while maintaining rapamycin. We resuspended the cells in warm SCD-hlta media with 1 ng/ul rapamycin to release the cells from G1. We collected cells every 10 min and extracted the genomic DNA using phenol-chloroform extraction. For qPCR, we chose four pairs of primers near ARS sequences (ARS416, ARS423, ARS428, and ARS-RT) and one pair of primers near Chr14-222k, a region that is far from any replication origins^65^. We ran all the qPCR on AriaMx Real-time PCR System (Agilent G8830A) and extracted the Ct values using the Agilent AriaMx Software (Version 1.71) (see Supplementary Table 2 for primer sequence and Supplementary Table 3 for raw qPCR data). We calculated the doses of the three regions relative to Chr14-222k based on the differences in their Ct values, and then corrected the bias for different primers by normalizing to *t* = 0 min. We averaged the values from different biological replicas, and calculated the average relative copy number $r$ for each ARS sequence at 30–40 min. We noticed a systematic drift in some of our dataset, i.e., all ARSs show lower copy numbers between 20 and 50 min, presumably due to the different efficiency of G1 release. We, therefore, normalized the relative copy number by the unperturbed ARSs: for CICI between ARS416 and ARS423 (ARS-RT), we normalized the relative copy number such that ${r}_{{{{{{\rm{ARS}}}}}}428}-{r}_{{{{{{\rm{ARS}}}}}}-{{{{{\rm{RT}}}}}}}({r}_{{{{{{\rm{ARS}}}}}}423})$ is the same as that of the background strain.

### Reporting summary

Further information on research design is available in the Nature Research Reporting Summary linked to this article.

## Supplementary information

Supplementary Information

List of supplementary files

Dataset 1

Dataset 2

Reporting Summary

## Data Availability

The data that support this study are available from the corresponding author upon reasonable request. The published Hi-C datasets used here include GSM1905067 and GSM1905068 available at GEO accession number GSE73890, and GSM2417264, GSM2417294, GSM2417295, GSM2417268, and GSM2417276 available at GEO accession number GSE90902, and GSM2327656, GSM2327657, GSM2327658 M-R1, and GSM2327659 available at GEO accession number GSE87311. Source Data are provided with this paper.

## Source data

Source data

## Code availability

The custom MATLAB codes used in this study are deposited into https://scholarsphere.psu.edu/ with the following links. For cell annotation program: https://scholarsphere.psu.edu/resources/17a9d031-ea32-436e-92ce-b4bd25a5d6cb. For the fluorescence dot detection program: https://scholarsphere.psu.edu/resources/87cf07a4-4bde-4a10-b9e7-13795aaccd67.
