# Supplementary material for: Chemically Induced Chromosomal Interaction (CICI) method to study chromosome dynamics and its biological roles
Source: Nat Commun. 2022 Feb 9;13:757. doi: 10.1038/s41467-022-28416-3 (PMC8828778; doi:10.1038/s41467-022-28416-3)
Supplement: Supplementary file 1 — Supplementary Information [file 41467_2022_28416_MOESM1_ESM.pdf]

Supplementary Information

This file includes:

Supplementary Figure 1-7

Supplementary Table 1-3

Separate files:

Supplementary Data 1-2

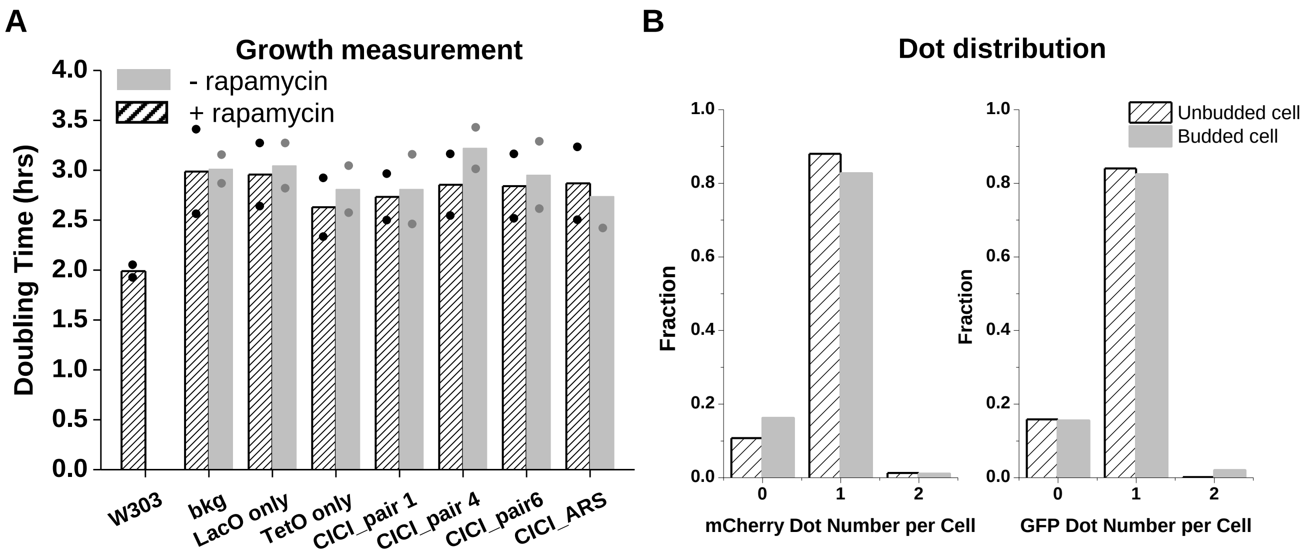

Supplementary Figure 1. Characterization of CICI and control strains. A) Doubling times of multiple strains, including wt strain (W303 a/alpha), rapamycin-resistant background (bkg), strains containing LacO and TetO but no LacI and TetR (LacO or TetO only), and four CICI strains (containing both arrays and the bound proteins). The rapamycin-resistant strains in general have comparable growth rates, regardless of the presence of rapamycin, LacO / TetO arrays, or different LacI / TetR fusions. Bar represents mean of two biological replica (individual data points are shown). **B)** Histograms of the number of LacI and TetR dots in unbudded and budded cells (only the dots in the mother cells are counted, total # of cells analyzed *N*=1168, cells that contain LacI dots *N* = 940, cells that contain TetR dots *N* = 902). We mostly observed a single dot, even in the budded cells, indicating that sister chromatids are mostly tethered together before one copy quickly moved into the daughter cells. 10-20% of cells contain no detectable dots, which is more likely due to imaging problems or transient dissociation of LacI-GFP / TetR-mCherry rather than a permanent loss of LacO / TetO arrays, because during time-lapse imaging, we often observe these cells regaining dots in later frames.

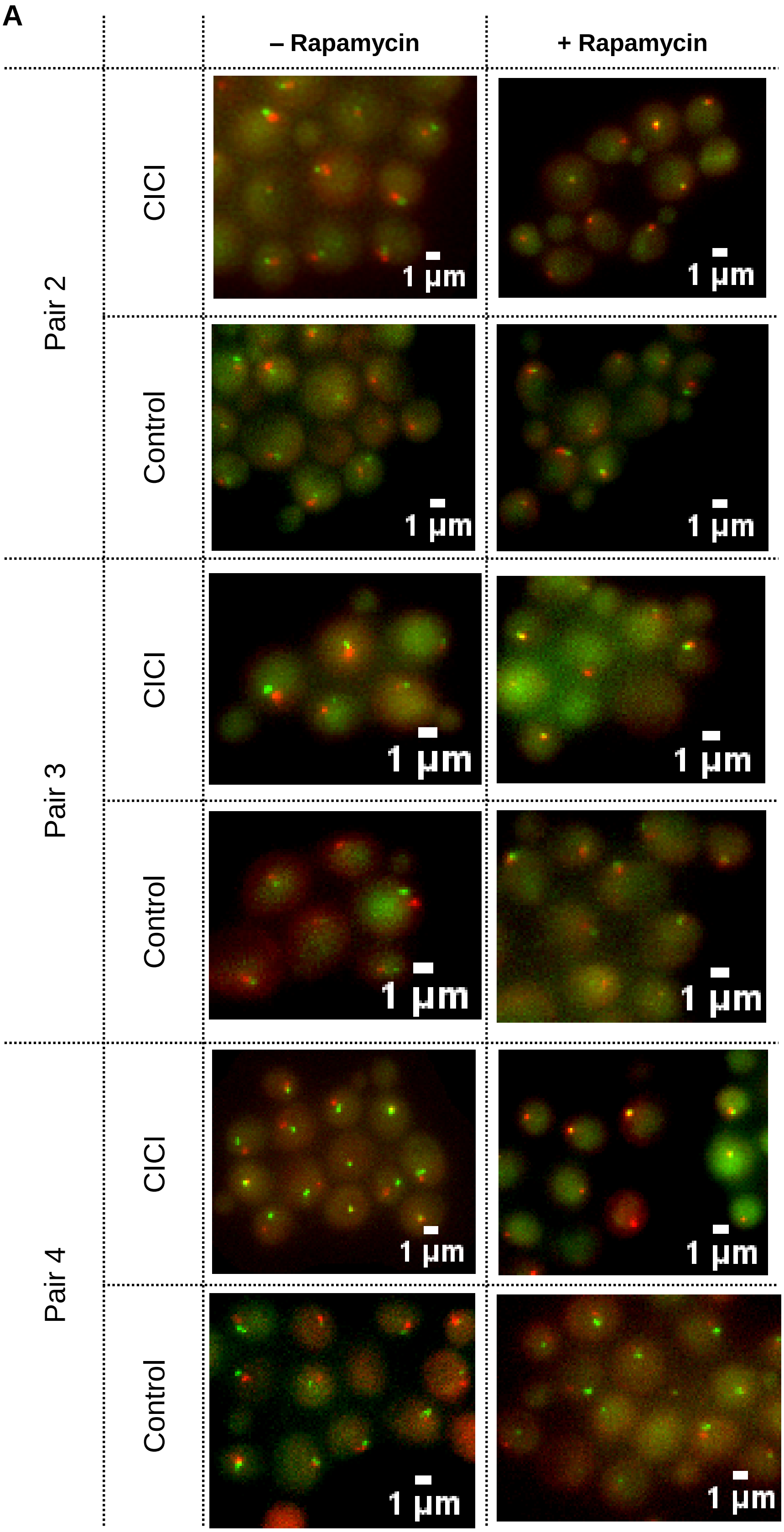
A
– Rapamycin
+ Rapamycin
Pair 2
ClCl
Control
Pair 3
ClCl
Control
Pair 4
ClCl
Control
1 µm

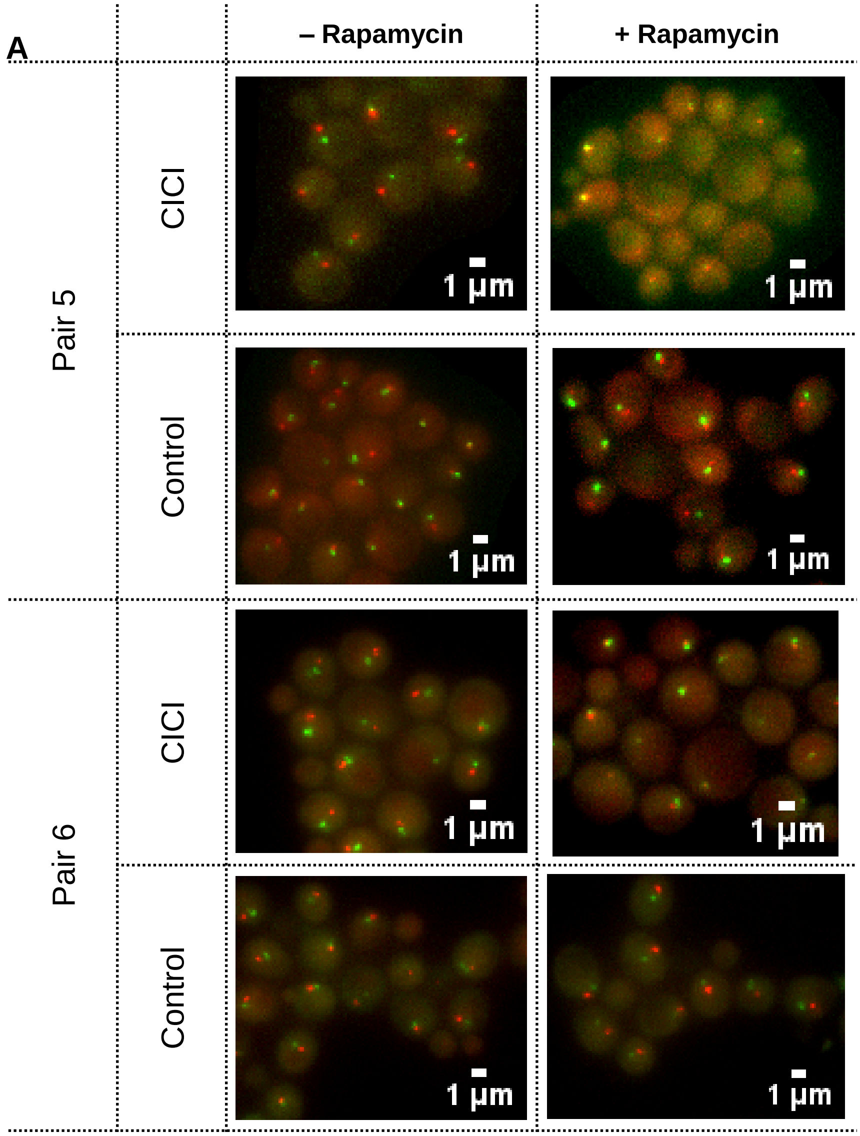

Supplementary Figure 2. Sample images and distance histograms. **A)** Sample images of ClCl and control strains containing pair 2-6 (the + rapamycin data represents 60min rapamycin incubation). **B)** Violin plot of initial distances of ClCl and ClCl control strains. White dot indicates sample median. The thick bar in the middle indicates the range from first quartile to third quartile, and the line extends from the bar to extra 1.5 inter-quartiles. Number of points for ClCl strain, pair 1-6: 116, 63, 70, 192, 205, 666; Number of points for control strain, pair 1-6: 407, 34, 90, 62, 117, 489. **C)** Same as B after 60min rapamycin incubation. Number of points for ClCl strain, pair 1-6: 46, 42, 63, 53, 258, 197; Number of points for control strain, pair 1-6: 100, 183, 99, 82, 10, 547.

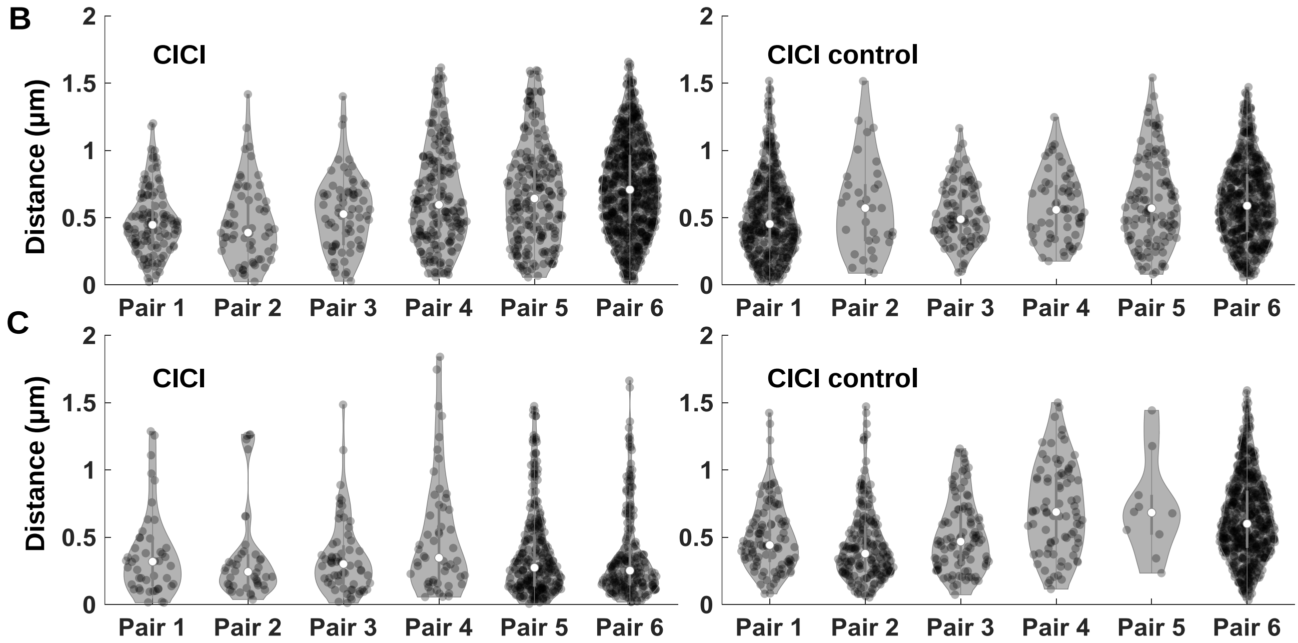

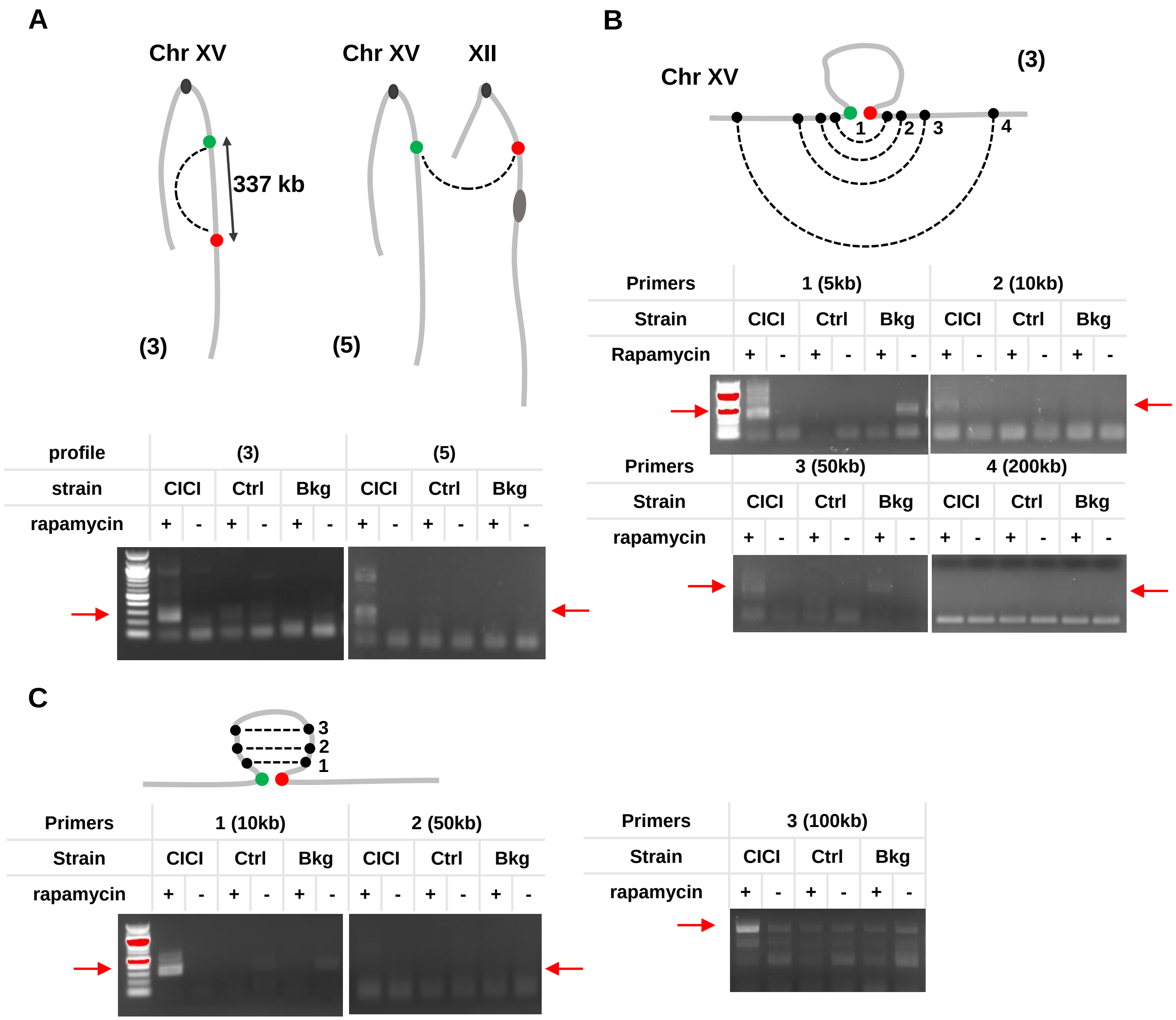

Supplementary Figure 3. 3C experiments confirm the ClCl-mediated contact formation. A) 3C signals of loci pairs 3 and 5 using primers immediately adjacent to the LacO / TetO arrays. We carried out the 3C measurement ± rapamycin using the ClCl strain, rapamycin-resistant background strain, and the -FKBP12/FRB control strain. 100bp ladder is shown on the left of the gel (same as in B & C). Red arrows represent the expected 3C PCR product. 3C signals are only visible in the ClCl strain +rapamycin. Same measurements were repeated at least twice and were shown to be reproducible (same as in B and C). **B)** Contacts outside the loop. 3C signals over four pairs of loci are examined. For pair 1, each primer is 5kb away from the LacO / TetO insertions sites, and this number is 10kb, 50kb, and 200kb for other tested 3C pairs. ClCl-induced 3C signals were detected for regions that are 5 and 10kb away, but become very weak at 50kb, and undetectable at 200kb. **C)** Contacts inside the loop. Three loci pairs that are 10kb, 50kb, and 100kb from LacO/TetO are examined. Increased 3C signals are detected at 10kb and 100kb.

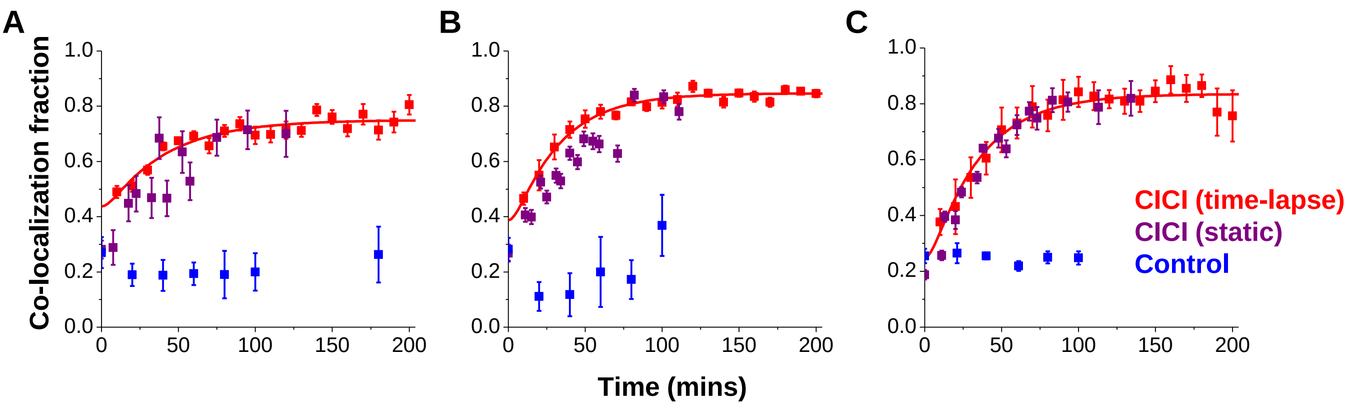

Supplementary Figure 4. Comparison of time-lapse and static measurement of CICI formation. A) Co-localization of CICI pair 4 as a function of time measured by time-lapse imaging (Total # of cells *N* = 1756). Fitting this data with the red curve yields CICI efficiency: 0.56 ± 0.19 and $t_{1/2}$: 27 min ± 1.3. These numbers are similar to the ones we obtained from the static measurements: CICI efficiency: 0.69 ± 0.09, and $t_{1/2}$: 37 ± 9 min. The static measurements of the control is shown in blue. Error bars represent standard error (same for B and C). **B)** Same as A) but for pair 5 (*N* = 1168 for time-lapse measurements). The parameters from the time-lapse measurements: CICI efficiency 0.75 ± 0.12, and $t_{1/2}$ = 21 ± 3 min. The ones from the static measurements: 0.81 ± 0.10, and $t_{1/2}$: 33 ± 11 min. **C)** Same as A) but for pair #6 (*N* = 1541 for time-lapse measurements). The parameters from the time-lapse measurements: CICI efficiency 0.78 ± 0.23, and $t_{1/2}$ = 22 ± 3 min. The ones from the static measurements: 0.78 ± 0.06, and $t_{1/2}$: 21 ± 3.5 min.

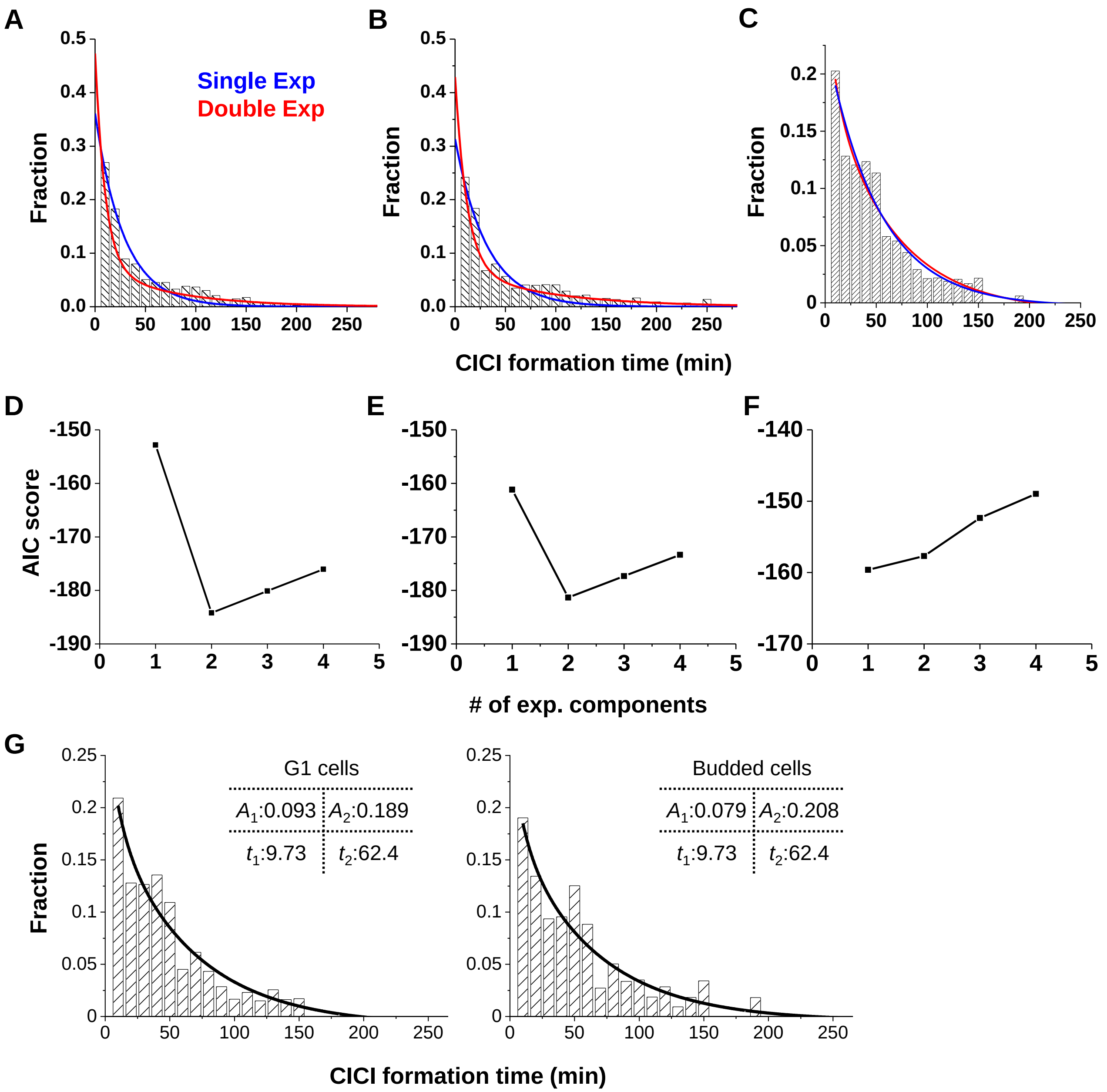

Supplementary Figure 5. Distribution of CICI formation time fits better with a double exponential function. A-C) Histogram of CICI formation time, as well as the single (blue) and double (red) exponential fit. The three panels are for CICI pair 5 (A), 4 (B), and 6 (C) (total # were 773, 378 and 524). **D - F)** AIC score of exponential fitting with different number of components for the histogram in A, B, and C. Double exponential function yields the best fit (lowest AIC score) in A&B, but has no advantage in C. **G)** CICI formation time for pair 6 in G1 vs budded cells (total # were 371 and 145 for G1 and budded cells, respectively). The slow component is mildly increased in the budded cell population.

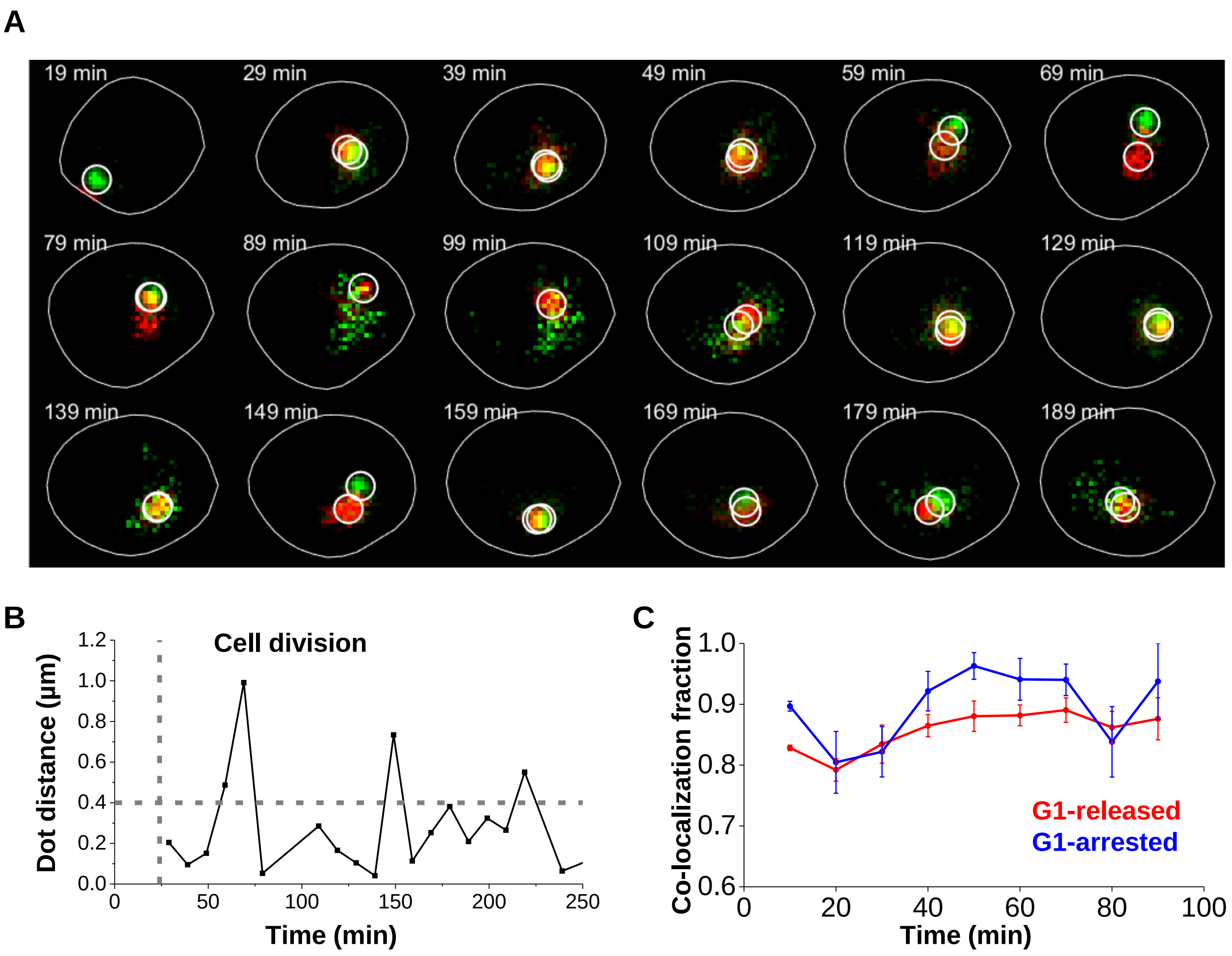

Supplementary Figure 6. CICI disruption in cell stages other than anaphase. A) Example time-lapse images of CICI strain (pair 5) that show CICI disruption after cell division. After cell division between 19-29 min, the co-localization of the two dots seem be disrupted at a few time points, one at 59 min and the other at 149 min. **B)** Distance between LacO and TetO arrays as a function of time for the cell in A. **C)** CICI co-localization fraction in G1-released vs G1-arrested cells (total number of cells: N = 705 and 2468 for G1-released and G1-arrested cells, respectively). The cells were arrested in G1 by α-factor and treated by rapamycin for ~1.5 hrs. At 0 min, we washed the cells using media ± α-factor to either continue G1 arrest (blue curve) or release from G1 (red curve). The released cells soon started budding (entering S phase), but the co-localization between the two dots did not drop below the G1 level, indicating that replication does not significantly disrupt CICI contacts. Error bars represent standard error.

A

Consensus: 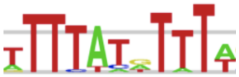

ARS	Sequence	Motif Score
ARS416	TTTTATGTTTA	13.5
ARS423	TTTTACATTTT	13.0
ARS428	TTTTATATTTT	13.3
ARS-RT	TTTTATGTTTA	15.2

B

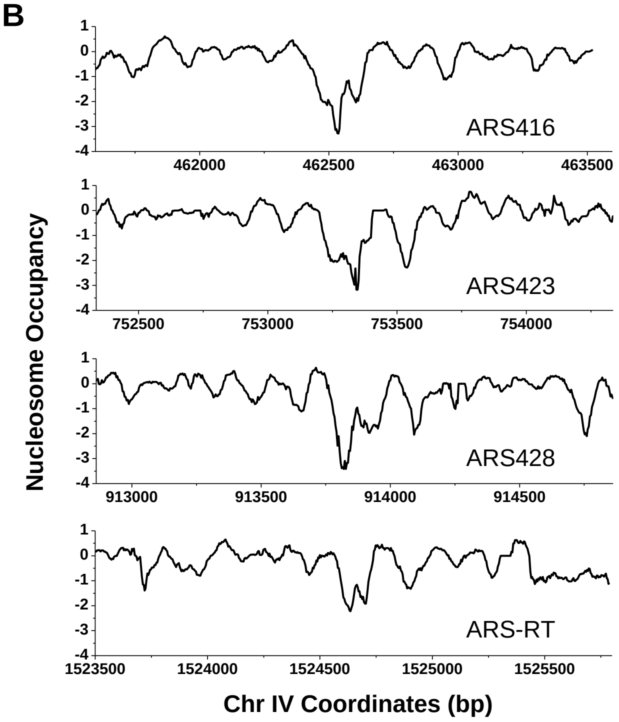

C

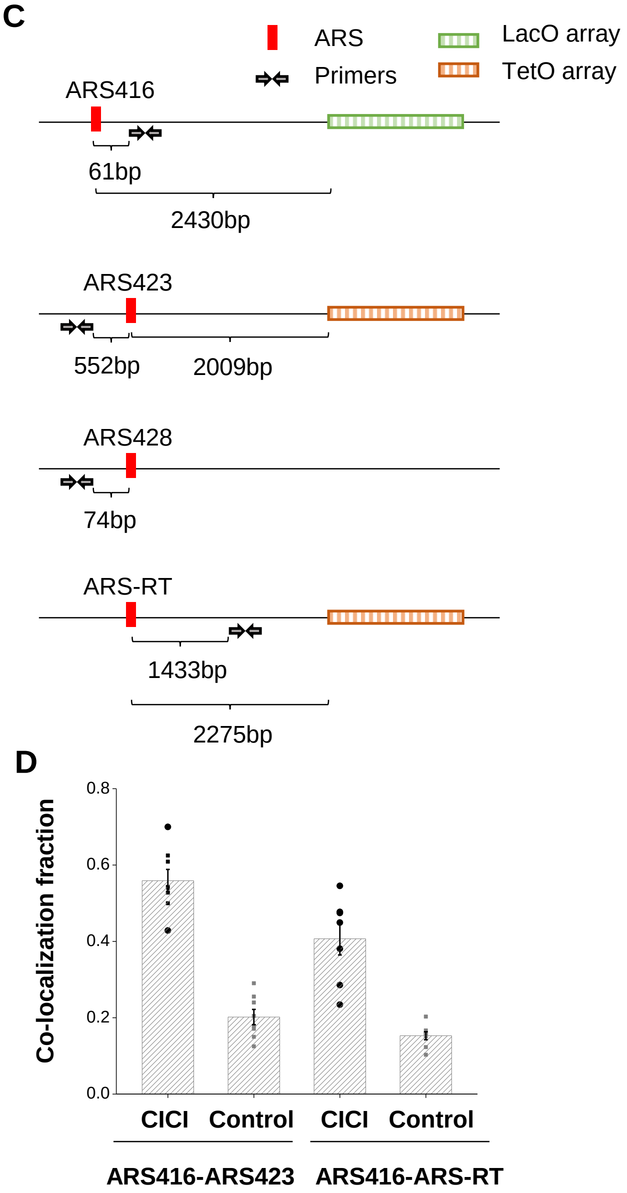

Supplementary Figure 7. Construction of the CICI strains near ARSs. A) The consensus sequences of ARS416, ARS423, ARS428, and ARS-RT, and the corresponding motif scores. **B)** Nucleosome occupancy near the three ARSs, all of which show local nucleosome depletion to comparable level. Data are from Lee et al. [48]. **C)** The genetic constructs of the strains. The locations of the qPCR probes for the measurement of DNA copy number (black arrows), LacO array near ARS416, and TetO array near ARS423 or ARS-RT are shown. **D)** Co-localization of the LacO and TetO dots in G1 arrested CICI or control cells after 1hr of rapamycin incubation (total number of cells: N = 212 (CICI) and 512 (control) for ARS416-ARS423 pair, 559 (CICI) and 628 (control) for ARS416-ARS-RT pair). Error bars represent standard error of the fraction.

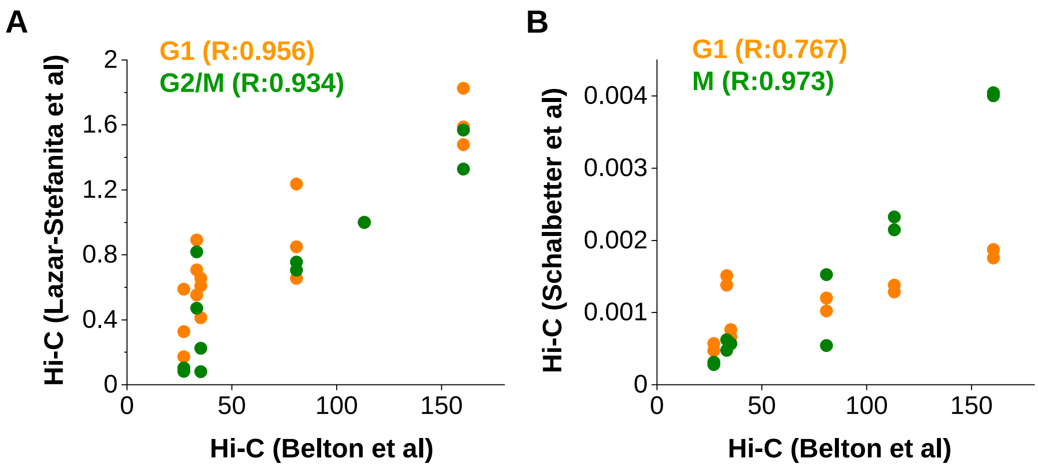

Supplementary Figure 8. Comparison of Hi-C data used in the manuscript (from Belton et al.) with two more datasets, Lazar-Stefanita et al. and Schalbetter et al. Belton et al. dataset was measured in asynchronized cell population, while the latter two were carried out in synchronized cells. For Lazar-Stefanita et al, we analyzed the G1 and G2/M data (nocodazole arrest) (biological replica N = 3 and 2, respectively). For Schalbetter et al., we analyzed G1 and M data (N = 2 for both datasets). Hi-C contact frequencies of the six CICI pairs in Fig 2 measured in Belton et al. (x axis) is plotted against those measured in Lazar-Stefanita et al. (A) and Schalbetter et al. (B). “R” represents Pearson correlation. Data points from individual experiments are shown. The raw Hi-C values are also presented in Table S5.

Supplementary Table 1

Plasmid	Note	Note
pSR13	256X LacO with a LEU2 marker	Rohner et al.
pSR11	192X TetO with a TRP1 marker	
pLB49_K1	Unique sequences K1 flanking an URA3 selection marker	This study
pLB49_K2	Unique sequences K2 flanking an URA3 selection marker	
pMY48	*REV1pr* -LacI-FKBP12-*REV1pr* -TetR-FRB with an ADE2 marker	
pMY49	*REV1pr* -LacI-*REV1pr* -TetR with an ADE2 marker	
pMY63	*REV1pr* -LacI-GFP-*REV1pr* -TetR-mCherry with a HIS3 marker	
pMY75	*GAL10* -HO with an URA3 marker	

Strain	Genotype	Note
yLB109	MATa, *tor1-1, fpr::NAT, bar1*	background strain
yMD215	MATa, *tor1-1, fpr::NAT, bar1, HIS3::REV1pr-tetR-mCherry-REV1pr-LacI-GFP, ADE2::REV1pr-LacI-FKBP12-REV1pr-TetR-FRB*	
yMD216	MATa, *tor1-1, fpr::NAT, bar1, HIS3::REV1pr-tetR-mCherry-REV1pr-LacI-GFP, ADE2::REV1pr-LacI-REV1pr-tetR*	
yMD251	MATa, *tor1-1, fpr::NAT, bar1, HIS3::REV1pr-tetR-mCherry-REV1pr-LacI-GFP, ADE2::REV1pr-LacI-FKBP12-REV1pr-TetR-FRB, Chr15::433495_LacO, Chr15::214015_TetO*	100kb_CICI
yMD259	MATa, *tor1-1, fpr::NAT, bar1, HIS3::REV1pr-tetR-mCherry-REV1pr-LacI-GFP, ADE2::REV1pr-LacI-REV1pr-tetR, Chr15::433495_LacO, Chr15::214015_TetO*	100kb_Control
yMD266	MATa, *tor1-1, fpr::NAT, bar1, HIS3::REV1pr-tetR-mCherry-REV1pr-LacI-GFP, ADE2::REV1pr-LacI-FKBP12-REV1pr-TetR-FRB, Chr15::497047_LacO, Chr15::150277_TetO*	150kb_CICI
yMD249	MATa, *tor1-1, fpr::NAT, bar1, HIS3::REV1pr-tetR-mCherry-REV1pr-LacI-GFP, ADE2::REV1pr-LacI-REV1pr-tetR, Chr15::497047_LacO, Chr15::150277_TetO*	150kb_Control
yMD292	MATa, *tor1-1, fpr::NAT, bar1, HIS3::REV1pr-tetR-mCherry-REV1pr-LacI-GFP, ADE2::REV1pr-LacI-FKBP12-REV1pr-TetR-FRB, Chr15::497047_LacO, Chr15::834487_TetO*	300kb_CICI
yMD291	MATa, *tor1-1, fpr::NAT, bar1, HIS3::REV1pr-tetR-mCherry-REV1pr-LacI-GFP, ADE2::REV1pr-LacI-REV1pr-tetR, Chr15::497047_LacO, Chr15::834487_TetO*	300kb_Control
yMD282	MATa, *tor1-1, fpr::NAT, bar1, HIS3::REV1pr-tetR-mCherry-REV1pr-LacI-GFP, ADE2::REV1pr-LacI-FKBP12-REV1pr-TetR-FRB, Chr12::616331_LacO, Chr12::306772_TetO*	Chr12_CICI
yMD283	MATa, *tor1-1, fpr::NAT, bar1, HIS3::REV1pr-tetR-mCherry-REV1pr-LacI-GFP, ADE2::REV1pr-LacI-REV1pr-tetR, Chr12::616331_LacO, Chr12::306772_TetO*	Chr12_Control
yMD274	MATa, *tor1-1, fpr::NAT, bar1, HIS3::REV1pr-tetR-mCherry-REV1pr-LacI-GFP, ADE2::REV1pr-LacI-FKBP12-REV1pr-TetR-FRB, Chr15::497047_LacO, Chr12:306772_TetO*	inter_CICI (1)
yMD275	MATa, *tor1-1, fpr::NAT, bar1, HIS3::REV1pr-tetR-mCherry-REV1pr-LacI-GFP, ADE2::REV1pr-LacI-REV1pr-tetR, Chr15::497047_LacO, Chr12:306772_TetO*	inter_Control (1)
yMD320	MATa, *tor1-1, fpr::NAT, bar1, HMR::HMRα-B, GAL10::GAL10pr-HO, HIS3::REV1pr-tetR-mCherry-REV1pr-LacI-GFP, ADE2::REV1pr-LacI-FKBP12-REV1pr-TetR-FRB, MAT::206345_LacO, HMR::299404_TetO*	MAT_CICI

yMD323	MAT<u>a</u>, *tor1-1, fpr::NAT, bar1, HMR::HMRα-B, GAL10::GAL10pr-HO, HIS3::REV1pr-tetR-mCherry-REV1pr-LacI-GFP, ADE2::REV1pr-LacI-REV1pr-TetR, MAT::206345_LacO, HMR::299404_TetO*	MAT_Control
yFZ43	MAT<u>a</u>, *tor1-1, fpr::NAT, bar1, HIS3::REV1pr-tetR-mCherry-REV1pr-LacI-GFP, ADE2::REV1pr-LacI-FKBP12-REV1pr-TetR-FRB, Chr4::465035_LacO, Chr4::1522313_TetO*	ARS416-ARS_RT
yFZ44	MAT<u>a</u>, *tor1-1, fpr::NAT, bar1, HIS3::REV1pr-tetR-mCherry-REV1pr-LacI-GFP, ADE2::REV1pr-LacI-REV1pr-tetR, Chr4::465035_LacO, Chr4::1522313_TetO*	ARS416-ARS_RT control
yFZ45	MAT<u>a</u>, *tor1-1, fpr::NAT, bar1, HIS3::REV1pr-tetR-mCherry-REV1pr-LacI-GFP, ADE2::REV1pr-LacI-FKBP12-REV1pr-TetR-FRB, Chr4::465035_LacO, Chr4:755413_TetO*	ARS416-ARS423
yFZ46	MAT<u>a</u>, *tor1-1, fpr::NAT, bar1, HIS3::REV1pr-tetR-mCherry-REV1pr-LacI-GFP, ADE2::REV1pr-LacI-REV1pr-tetR, Chr4::465035_LacO, Chr4:755413_TetO*	ARS416-ARS423 control
yFZ47	MAT<u>a</u>, *tor1-1, fpr::NAT, bar1, HIS3::REV1pr-tetR-mCherry-REV1pr-LacI-GFP, ADE2::REV1pr-LacI-FKBP12-REV1pr-TetR-FRB, Chr4::465035_LacO, Chr4:916082_TetO*	ARS416-ARS428
yFZ48	MAT<u>a</u>, *tor1-1, fpr::NAT, bar1, HIS3::REV1pr-tetR-mCherry-REV1pr-LacI-GFP, ADE2::REV1pr-LacI-REV1pr-tetR, Chr4::465035_LacO, Chr4:916082_TetO*	ARS416-ARS428 control
yFZ78	MAT<u>a</u>, *tor1-1, fpr::NAT, bar1, HIS3::REV1pr-tetR-mCherry-REV1pr-LacI-GFP, ADE2::REV1pr-LacI-FKBP12-REV1pr-TetR-FRB, Chr15::433495_LacO, Chr4:916082_TetO*	inter_CICI (2)
yFZ77	MAT<u>a</u>, *tor1-1, fpr::NAT, bar1, HIS3::REV1pr-tetR-mCherry-REV1pr-LacI-GFP, ADE2::REV1pr-LacI-REV1pr-tetR, Chr15::433495_LacO, Chr4:916082_TetO*	inter_Control (2)

Supplementary Table 2

All primers are purchased from Integrated DNA Technologies

Primers for ARS416	
PF-ARS416	AGGAAAATATACATCGCAGGGG
PR-ARS416	GGTCATTGTAGCGTATGCG
Primers for ARS423	
PF3-ARS423	GTAAGGATGACATCTCAGTGGG
PR3-ARS423	GCACATCATTATAGTCTTGCATCC
Primers for ARS428	
PF-ARS428	CCACTTGAGAGAGGATCAAGG
PR-ARS428	CCACCGACTCTATAAGTACACAGC
Primers for ARS-RT	
PF3-ARS-RT	CACCAGCTGGTGAAGGAATTTG
PR3-ARS-RT	AGTGCTGATATAAGCCCTACTAGGAAG
Primers for Chr14-222k	
PF_Chr14_222k	ATCCTGCGCGTTGACATAA
PR_Chr14_222k	CAATTTTGCTCAACGTCTGCA
Primer for 3C measurement:	
P-F-CICIpair3	TACTGACGTTTCAGTAGTTAATCCAGAG
P-R-CICIpair3	GTAACTTTGGAAGACGCTCCCAC
P-F-CICIpair5	CATGAATACTTCCCAAAACAAACAAC
P-R-CICIpair5	CCAAAACTTGCTGTTCATATGACAAC
P-F-CICI-outerLoop1	ATCTAGTTTCTTGTCACCTCATACG
P-R-CICI-outerLoop1	CTCCTTCAATAGTTTATTTAGGGAAGC
P-F-CICI-outerLoop2	CACTTCATTGTTGTATAGAACCTCTGC
P-R-CICI-outerLoop2	GCGTAAACATTCAGAGAAGTACATGAC
P-F-CICI-outerLoop3	GTCATTGGTTGCAAAAACTAAACTCTAC
P-R-CICI-outerLoop3	CCTCTTTTGACTAGGCATCTGATAATG
P-F-CICI-outerLoop4	CCAACTGTTTATTGGGTACGATTCC
P-R-CICI-outerLoop4	AAGTCACATATCGAGTCGAAATCGG
P-F-CICI-innerLoop1	GCTCGTGCCATCACAATTAATATAAGG
P-R-CICI-innerLoop1	AATCGTGAAGAATTTCCAGATTTAACAG
P-F-CICI-innerLoop2	ACCTCAATTGGTACAAATGTATATCGG
P-R-CICI-innerLoop2	TGGTTTCTCAGCAGTTCTACTACTG
P-F-CICI-innerLoop3	GTTTACCGAACCATTTATGGTTCTC
P-R-CICI-innerLoop3	CACCTTTTACCAGCATTCTTGAAGG

Primer for DNA repair assay:

PF_MATa	GGGTATGTAATATGAGAATC
PF1-MAT-B	GCCCACTTCTAAGCTGATTTCAATCTCTCC
PR2-MAT-B	CCTGTTCTTAGCTTGTACCAGAGG
PMY-F1-ACT1	GGTAACGAAAGATTCAGAGCCCC
PMY-R1-ACT1	CGACATCACACTTCATGATGGAG

Supplementary Table 3

Biological replica #1

time (min)	CT values of ARS416			CT values of ARS423			CT values of ARS428			CT values of ARS-RT			CT values of Chr14-222k		
0	19.15	19.27	19.25	18.95	18.96	18.98	19.31	19.46	19.38	19.87	19.86	19.85	18.94	18.82	18.97
10	18.75	18.62	18.74	18.27	18.26	18.35	18.73	18.76	18.76	19.2	19.15	19.15	18.59	18.21	18.4
20	17.97	18.03	18.11	18.24	18.22	18.05	18.17	18.11	18.12	19.14	18.93	19.24	18.33	18.49	18.3
30	17.55	17.84	17.91	17.91	17.74	17.87	17.91	17.7	17.88	18.86	18.86	18.92	18.33	18.46	18.41
40	18.37	18.62	18.66	18.55	18.54	18.52	18.66	18.7	18.76	19.48	19.3	19.63	18.92	18.92	19.04
50	18.11	18.05	18.21	18.05	18.03	17.99	18.34	18.36	18.36	19.06	18.75	18.82	18.47	18.35	18.39
60	18.4	18.34	18.42	18.03	18	18.15	18.46	18.52	18.51	18.91	18.99	18.98	18.05	17.94	18.1
70	17.74	17.65	17.74	17.3	17.28	17.34	17.71	17.74	17.67	18.22	18.19	18.24	17.53	17.19	17.42
80	17.23	17.43	17.42	17.23	17.24	17.09	17.55	17.54	17.47	17.99	17.93	18.16	17.21	17.38	17.21

Biological replica #2

time (min)	CT values of ARS416			CT values of ARS423			CT values of ARS428			CT values of ARS-RT			CT values of Chr14-222k		
0	18.58	18.45	18.39	17.89	17.96	17.98	18.37	18.3	18.35	18.73	18.74	18.83	18.29	18.18	18.17
10	18.56	18.49	18.43	17.92	18	17.89	18.4	18.36	18.31	18.73	18.68	18.78	18.38	18.31	18.27
20	18.32	18.13	18.21	18.04	17.96	17.95	17.96	18.13	18.13	18.92	18.77	18.97	18.4	18.33	18.35
30	17.73	17.68	17.66	17.49	17.47	17.51	17.62	17.56	17.67	18.42	18.62	18.45	18.16	18.19	18.21
40	17.6	17.58	17.51	17.25	17.23	17.33	17.56	17.53	17.43	17.98	18.17	18.09	17.71	17.74	17.81
50	17.48	17.57	17.46	17.02	17.07	17.1	17.42	17.4	17.52	NaN	17.8	17.89	17.45	17.56	17.6
60	17.29	17.55	17.68	17.17	17.13	17.24	17.71	17.46	17.7	18.05	18.09	18.12	17.43	17.6	17.77
70	16.74	16.94	16.98	16.76	16.74	16.7	16.99	16.97	16.93	17.36	17.1	17.5	16.95	16.96	NaN
80	16.89	16.85	16.99	16.84	16.87	16.83	17.11	17.08	17.07	17.47	17.26	17.23	17.08	17	NaN

Biological replica #3

time (min)	CT values of ARS416			CT values of ARS423			CT values of ARS428			CT values of ARS-RT			CT values of Chr14-222k		
0	18.14	18.21	18.33	17.9	17.87	17.89	18.26	18.39	18.36	18.66	18.67	18.66	17.78	17.67	17.83
10	18.28	18.21	18.29	17.92	17.9	17.92	18.29	18.38	18.36	18.72	18.66	18.66	18.2	17.89	17.94
20	17.74	17.85	17.94	17.78	17.74	17.59	18.06	18.04	18.09	18.52	18.46	18.61	17.75	17.86	17.7
30	17.41	17.65	17.76	17.55	17.56	17.7	17.7	17.48	17.69	18.4	18.45	18.5	17.79	17.91	17.88
40	17.19	17.46	17.42	17.43	17.35	17.34	17.38	17.45	17.42	18.11	17.89	18.31	17.83	17.8	17.93
50	17.1	17.1	17.26	17.04	17.1	17.03	17.3	17.33	17.24	17.94	17.62	17.69	17.47	17.47	17.4
60	17.38	17.36	17.36	17.07	17.05	17	17.29	17.17	17.34	17.81	17.94	17.83	17.47	17.52	17.56
70	17.39	17.31	17.25	17.06	17	17.05	17.24	17.19	17.17	17.72	17.85	17.79	17.33	17.34	17.45
80	17.32	17.34	17.19	16.99	17.02	17.05	17.26	17.17	17.34	17.76	17.68	17.73	17.2	17.26	17.34
