# Supplementary material for: Chemically Induced Chromosomal Interaction (CICI) method to study chromosome dynamics and its biological roles
Source: Nat Commun. 2022 Feb 9;13:757. doi: 10.1038/s41467-022-28416-3 (PMC8828778; doi:10.1038/s41467-022-28416-3)
Supplement: Supplementary file 2 — List of supplementary files [file 41467_2022_28416_MOESM2_ESM.docx]

Supplementary Data 1: Hi-C data between LacO / TetO insertions sites from published datasets.

Supplementary Data 2: Distance between LacO and TetO in CICI and control cells at different time points of rapamycin addition.
